# Characterization and complete genome sequences of two novel variants of the family *Closteroviridae* from Chinese kiwifruit

**DOI:** 10.1371/journal.pone.0242362

**Published:** 2020-11-23

**Authors:** Xin Feng, Rui-lian Lai, Min-xia Gao, Wen-guang Chen, Ru-jian Wu, Chun-zhen Cheng, Yi-ting Chen

**Affiliations:** 1 Fruit Research Institute, Fujian Academy of Agricultural Sciences, Fuzhou, Fujian, China; 2 Research Centre for Engineering Technology of Fujian Deciduous Fruits, Fuzhou, Fujian, China; 3 Fujian Agriculture and Forestry University, Fuzhou, Fujian, China; Deen Dayal Upadhyaya Gorakhpur University, INDIA

## Abstract

Two distinct closterovirus-like genome sequences (termed AdV-1 v1 and v2) were identified in *Actinidia chinensis* var. *deliciosa* ‘Miliang-1’ that had no disease symptoms using high-throughput sequencing. Using overlapping reverse transcription-polymerase chain reaction and rapid amplification of cDNA ends, the genomic sequences of AdV-1 v1 and v2 were confirmed as 17,646 and 18,578 nucleotides in length, respectively. The two complete genomes contained 9 and 15 open reading frames, respectively, coding for proteins having domains typical of *Closteroviridae*, such as RNA-dependent RNA polymerase (RdRp), heat shock protein 70 homolog (HSP70h) and coat protein (CP). Sequence analysis showed that the amino acid sequences of RdRp, HSP70h, and CP of the two variants exhibited high similarity (> 80%), while their genomic organization was somewhat different. This suggested that the two viral genomes identified here are variants of the family *Closteroviridae* in a single kiwifruit host. Furthermore, phylogenetic relationship analysis revealed that the two variants had a closer relationship with the unclassified virus *Persimmon virus* B (PeVB) and *Actinidia virus* 1 (AcV-1) than with other members of the family *Closteroviridae*, as did their genomic organization. It is speculated that the two variants, together with PeVB and AcV-1 belong to a new subfamily of *Closteroviridae*.

## Introduction

Kiwifruit is a perennial vine plant that began domestication and cultivation at the beginning of the 20th century [1]. It is currently grown principally in China, New Zealand, Chile, Italy, and Greece [2]. Kiwifruit is greatly appreciated by consumers for its high nutritional value and its health benefits. Over the last ten years, the global area of kiwifruit cultivation has increased by 71.9%, and 75% in China according to the Food and Agriculture Organization (FAO). However, this increase means that the spread of diseases is increasingly serious.

Viral diseases in plants have a long incubation period, and therefore represent a significant hidden danger during production. In 2003, New Zealand researchers discovered for the first time the symptoms of viral infection caused by *Apple stem grooving virus* in a number of kiwifruit seedlings imported from China [3]. Subsequently, sixteen kiwifruit viruses have been identified worldwide, including eight that are non-specific: *Apple stem grooving virus*, *Actinidia virus* X, *Alfalfa mosaic virus*, *Cucumber necrosis virus*, *Cucumber mosaic virus*, *Ribgrass mosaic virus*, *Turnip vein clearing virus* and *T*omato *necrotic spot associated virus* [3–6], six specific kiwifruit viruses: *Actinidia virus* A, *Actinidia virus* B, *Actinidia citrivirus*, *Actinidia virus* 1, *Actinidia chlorotic ringspot-associated virus* and *Actinidia seed-borne latent virus* [7–10], and two pathogenic viruses (*Cherry leaf roll virus* and *Pelargonium zonate spot virus*) [4]. These known kiwifruit viruses belong to different families, including *Betaflexiviridae*, *Bromoviridae*, *Virgaviridae*, *Tombusviridae*, *Bunyaviridae*, *Secoviridae*, and *Closteroviridae*.

In 2015, a deep-sequencing analysis was conducted to identify long-noncoding RNAs in kiwifruit grown in China by our laboratory. Interestingly, two new putative viral genome sequences that shared significant sequence similarity with viruses classified as belonging to the family *Closteroviridae*, were detected. The family *Closteroviridae* consists of single-stranded and long positive-sense RNA genomes, and is categorized into four genera: *Closterovirus*, *Crinivirus*, *Ampelovirus* and *Velarivirus*, according to their large differences in molecular and biological characteristics [11]. In addition, this family also includes a number of unassigned members because of ambiguous phylogenies or a lack of genetic data and vectors, such as *Bulueberry virus* A and *Mint vein banding virus* [11, 12].

In the present study, the two new putative viral genome sequences were confirmed using overlapping reverse transcription-polymerase chain reaction (RT-PCR) and rapid amplification of cDNA ends (RACE-PCR). Their genomic structure and organization were then analyzed and compared with other members of the family *Closteroviridae*. Finally, three phylogenetic trees were constructed based on the amino acid sequences of three typical viral proteins (RNA-dependent RNA polymerase, heat shock protein 70 homolog, and coat protein) to elucidate their phylogenetic relationship. To the best of our knowledge, this is the first report of variant genome sequences of *Closteroviridae* from Chinese kiwifruit, which should represent the basis for future studies of their pathogenesis.

## Materials and methods

### RNA preparation and deep-sequencing

In September 2015, fruits with total soluble solids of 6.0–7.0% were collected from 4-year old kiwi plants (*Actinidia chinensis* var. *deliciosa* ‘Miliang-1’) that displayed no disease symptoms from an orchard in Gaozhen Village, Xikou Town, Jianning County, Sanming City, Fujian Province, China. Total RNA was extracted using an RNAprep Pure Plant kit (Tiangen, Beijing, China). RNA contamination and integrity were monitored using 1.0% agarose gel electrophoresis and an RNA Nano 6000 assay kit for the Bioanalyzer 2100 system (Agilent Technologies, CA, USA). A NanoPhotometer^®^ spectrophotometer (Implen, CA, USA) was used to estimate RNA purity, and a Qubit^®^ RNA assay kit used to measure the RNA concentration in a Qubit^®^ 2.0 Flurometer (Life Technologies, CA, USA).

High-quality RNA was used for sequencing. Firstly, ribosomal RNA (rRNA) was removed using an Epicentre Ribo-zero^™^ rRNA removal kit (Epicentre, USA), the rRNA-free residues cleaned using ethanol precipitation. Sequencing libraries were then generated using the rRNA-depleted RNA using a NEBNext^®^ Ultra^™^ Directional RNA Library prep kit for the Illumina^®^ platform (NEB, USA), in accordance with the manufacturer’s instructions. Following the assessment of library quality, a cBot Cluster Generation System was used to cluster the index-coded samples using a TruSeq PE cluster kit v3-cBot-HS (Illumia), in accordance with the manufacturer’s recommendations. The libraries were then sequenced on an Illumina HiseqX10 platform from which 150 bp paired-end reads were generated.

After removing reads containing adapters, ploy-N, and low-quality reads, the high-quality reads were *de novo* assembled into large contigs using Trinity software [13]. Assembled contigs were then mapped to the kiwifruit reference genome [14]. The un-mapped contigs were further annotated with pafm, GO and KEGG databases to remove host contigs. The retained contigs were searched against viral genome/protein sequences (taxid:10239) in the National Center for Biotechnology Information Database (NCBI, https://blast.ncbi.nlm.nih.gov/) using BLASTN (nucleotide BLAST) and BLASTX (translated nucleotide BLAST) applications.

### Primer design and amplification

To confirm the results of next-generation sequencing, high-quality RNAs extracted from mixed samples of leaves, stems and fruits of *Actinidia chinensis* var. *deliciosa* ‘Miliang-1’ were reverse transcribed (RT) using a SMART^™^ RACE cDNA Amplification kit (Takara, Dalian, China) for reverse transcription-polymerase chain reaction (RT-PCR) and 5’/3’-rapid amplification of cDNA ends (RACE). From the two contig sequences obtained from deep-sequencing, 12 to 16 pairs of specific primers were designed for RT-PCR using DNAMAN software. The detailed RT-PCR primer sequences and fragment positions are displayed in Table 1. Subsequently, based on sequences derived from the RT-PCR results, specific primers were designed for 5’ and 3’ RACE-PCR to obtain sequences of the 5’/3’-termini of the two viruses. The detailed primer sequences, annealing temperatures, and sizes are summarized in S1 Table.

**Table 1:** Oligonucleotide primers used for overlapping RT-PCR amplification to determine nucleotide sequences of the AdV-1 variants.

AdV-1	Forward primer (5′-3′)	Reverse primer (5′-3′)	Fragment position^a^
**v1**	1F: TTGATCGTGTGCTCTGGAGT	1R: TGACGGTTGCGATCGATCTA	133–2374
	2F: CGTAGATTGCGCCGAAAGAA	2R: CCCCTCTCCATCACCGTATC	1679–3691
	3F: GGTGGCCATGTATTTCGGTC	3R: CATATCTGCCGCTTTCCCAC	2866–5198
	4F: CATGATATAGCCATATCTCAGGAG	4R: CACTGAACAATTCGTCCAGTAATTC	4637–6725
	5F: CGGTGTTGGAGAGTGTAGGT	5R: AAGTCGGTGGTGGTGTCTAG	6120–8409
	6F: ATTCGGAAAGGCGGTTATGC	6R: TTCCGACGGTCATGTTCTCA	7385–9785
	7F: TGTTCAGCTCAGTCGACCAT	7R: GGATATCCCGGACAGCGTAA	9408–11301
	8F: ACTGGACGCCTCTTCTTTGA	8R: AACCGTCACCACCCAGTTAT	10169–11968
	9F: GACGTGCAACAACATCTCGT	9R: CCTCTGGGTATCAACGGTGT	11695–13931
	10F: GAAGAGTGTTGGCGGATGTC	10R: TTGGCCGTAAAGACACAACG	13643–15902
	11F: CAGCGTTATCCCAGCAGTTC	11R: CTCTCAGGGTTTGGATGGGT	14516–16967
	12F: CACGGCAAGTTTAATTCGACTAC	12R: CATCTTATCATCGATGATAATACG	16413–17646
**v2**	21F: AACCCACTCAGTTCCTCAGG	21R: TTTTAACGTCCTGTGCTCGC	1–1582
	22F: GAGCCGTTTGATGATGCTCC	22R: ACACTCCATGGTCCACAGTT	958–2483
	23F: GGTGTCATCTCTCCCGGATT	23R: GAAGACACAGAAGCAGCCAG	1282–2997
	24F: GTCGAGTGTCCTGGGAGAAG	24R: GCAGAGTCTTTCAACTGGGC	2173–3870
	25F: AACATAGTCAGCTGGCCAGT	25R: AGTCGTCTATGGCAGCCTTT	3273–5111
	26F: ATAAGGACGTTGACAGCCCA	26R: ACCGCCAATAGACCAAGACA	3425–5828
	27F: GTCAGGGAAGGGTCAAGACA	27R: GCAAAACCACGAGCCTGTAA	5400–7507
	28F: TTTGATGTGGACCTGTCGGA	28R: ACCCTAGCTTCTACGTGACG	6625–9011
	29F: GGATGGGCGATGTGTACAAC	29R: CGTGTTAGCTGTGCCTGATC	7979–10432
	30F:ACTTGACGCTTCATCATTGACTAAG	30R: ATACATCTTCATGCTCGTGACTTC	10012–11120
	31F: GATCAGGCACAGCTAACACG	31R: GTTGTAAGCGCCAAGTTCGA	10413–12900
	32F: ACTGGAAGTGGTCGATGGTT	32R: TGTGACGCAAAAGAACCCTG	11433–13737
	33F: TAAGGACAATGATGTGTCGCAAC	33R: CTTACGAGTAACGTGCTCATTTG	13326–14262
	34F: GCGTCACACCGTTAATACCC	34R: GTTGCCCAATTCAGTGACGT	13730–15845
	35F: TAGTGGATATGACGAGGCTTGAG	35R: TAGCCACACTAGGAGCTGACG	15447–17019
	36F: ATAAGGCGCTCAGAGGATCC	36R: GGAGGCCACAACAGTCAAAA	16537–18558

^a^ Nucleotide positions in the nucleotide sequences of the variants.

Both RT-PCR and RACE-PCR were performed in 200 μl-tubes in a 50 μl-reaction system containing 20 ng RT products, 0.4 μM forward/reverse primers and 25 μl DreamTaq Green Master Mix (2×; Thermo scientific, Calfornia, USA). The amplification program consisted of: initial PCR activation at 94°C for 3 mins, followed by 34 cycles of denaturation of 94°C for 30 s, primer annealing at 51.6 to 60°C (depending on the primers, as listed in Table 1 and S1 Table) for 30 s, primer extension at 72°C for 30 to 120 s (based on fragment size as listed in Table 1 and S1 Table), with a final extension at 72°C for 10 mins. The amplicons were then confirmed on 1.0% agarose gels and purified using an *EasyPure*^®^ Quick Gel extraction kit (Transgen, Beijing, China) in accordance with the manufacturer’s protocol, then cloned using a pEASY^®^-T1 cloning kit (Transgen, Beijing, China). At least three clones were sequenced by Beijing Liuhe Huada Gene Technology Co., Ltd (China).

### Viral sequence analysis

Nucleotide sequences of the viruses were analyzed using DNAMAN software, and their predicted open reading frames (ORFs) determined using Open Reading Frame Finder (https://www.ncbi.nlm.nih.gov/orffinder/). Pairwise comparisons of the full-length nucleotide sequences/amino acid sequences of viruses were performed using fast alignment in DNAMAN software with default parameters. Multiple sequence alignments were performed with complete alignment using ClustalX software using default parameters [15]. Molecular masses were calculated using the ProtParam tool (https://web.expasy.org/protparam/). Conserved domains and motifs were analyzed using the NCBI Blast application (https://blast.ncbi.nlm.nih.gov/Blast.cgi) and pfam (http://pfam.xfam.org/), respectively. Transmembrane helices of proteins were analyzed in ExPASy using the TMpred application (https://embnet.vital-it.ch/software/TMPRED_form.html). Amino acid sequences were used as input sequences to perform alignments using ClustalW in MEGA 10.1.8, from which phylogenetic trees were constructed using a neighbor-joining algorithm with Poisson correction, pairwise gap deletion and bootstrap with 1000 replicates [16].

## Results

### Sequence assembly and confirmation

A total of 96,299,434 clean reads were obtained by next-generation sequencing. These clean reads were then *de novo* assembled into larger contigs using the Trinity software, and host contigs were removed by mapping to the kiwifruit reference genome and annotated with pfam, GO and KEGG databases. Two un-mapped contigs with a length of 18,840 nucleotides (nt), and 19,7431 nt were retained. A BLASTN search of the NCBI database revealed that they had high sequence similarity to viral genome sequences. An additional BLASTX search demonstrated that both the two contigs contained ORFs that coded for three proteins with typical domains of *Closteroviridae*, such as RNA-dependent RNA polymerase, heat shock protein 70 homolog and coat proteins.

To confirm the sequences of the two putative *Closteroviridae* viruses, overlapping RT-PCR was performed using 12 and 16 specific primer pairs (Table 1). Primers were designed based on the nucleotide sequences of the two un-mapped contigs. Subsequently, RACE-PCR was conducted to obtain sequences of their 5’-and 3’-termini using specific primers (S1 Table) designed from the results of RT-PCR. Sequencing results of RT-PCR and RACE-PCR confirmed *de novo* assembly, with genomes of the two viruses were confirmed to be 17,646 nt and 18,578 nt in length, respectively (S1 Appendix).

DNAMAN sequence alignments showed that the two new viruses shared 81.38% (S1 Fig) and 65.01% (S2 Fig) nucleotide sequence identity, respectively, with the known *Actinidia virus* 1 (AcV-1, KX857665) isolated from *Actinidia chinensis* Planch var. *chinensis* ‘Hort16A’ grown in Italy, a newly discovered member of the family *Closteroviridae* [17]. Furthermore, similarity analysis between the two viral sequences using DNAMAN software (Table 2) and ClustalX software (S2 Table), revealed that they shared high sequence identity. This suggests that the two viral sequences were derived from distinct variants of *Closteroviridae*, having homology with *Actinidia virus* 1. Since the two new variants were isolated from *A*. *chinensis* var. *deliciosa* ‘Miliang-1’ grown in China, they were provisionally termed *Actinidia deliciosa virus* 1 variant 1 (abbreviated to AdV-1 v1), and variant 2 (AdV-1 v2), respectively.

**Table 2:** Sequence identities of *Actinidia deliciosa virus* 1 variant 1 and 2 with other members of the family *Closteroviridae*.

Genus	GenBank no.	Virus and Acronym	Nucleotide sequence identity (%)^a^	Amino acid sequence identity (%)^b^			
				ORF1a	RdRp	Hsp70h	CP
Unassigned	-	Actinidia deliciosa virus1 variant2 (AdV-1 v2)	60.60	60.24	80.47	88.87	88.07
Unassigned	KX857665	Actinidia virus 1(AcV-1)	81.38	86.50	93.53	93.15	93.42
Unassigned	NC025967	Persimmon virus B variant 1 (PeVB v1)	28.34	17.76	37.75	33.28	21.25
Unassigned	AB923925	Persimmon virus B variant 2 (PeVB v2)	28.52	19.03	37.76	35.18	23.08
Closterovirus	BYU51931	Beet yellow stunt virus (BYSV)	17.43	5.99	33.92	26.58	19.11
Closterovirus	NC006944	Mint virus 1 (MV-1)	28.24	15.37	34.97	24.32	17.96
Closterovirus	KU883267	Citrus tristeza virus (CTV)	25.65	17.98	32.65	28.26	20.58
Closterovirus	NC027712	Tobacco virus 1 (ToV-1)	27.48	15.32	33.02	24.51	18.52
Closterovirus	NC_008585	Raspberry mottle virus (RMV)	26.03	15.00	34.84	28.26	20.58
Crinivirus	NC010560, EU191905	Bean yellow disorder virus (BYDV)	-	9.85	22.04	23.72	16.08
Crinivirus	NC005209, NC005210	Beet pseudo-yellows virus (BPYV)	-	9.23	24.66	22.84	13.72
Ampelovirus	AF414119	Pineapple mealybug wilt-associated virus 1 (PMWaV-1)	21.75	9.66	24.33	20.74	15.44
Ampelovirus	NC016509	Grapevine leafroll-associated virus 1 (GLRaV-1)	27.20	12.51	24.21	24.83	12.39
Velarivirus	HM588723	Cordyline virus 1 (CoV-1)	25.80	11.30	23.60	23.54	14.06
Velarivirus	HE588185	Grapevine leafroll-associated virus 7(GLRaV-7)	28.25	12.11	20.95	25.08	14.29

^a.^ Nucleotide sequence alignment was performed using DNAMAN software using fast alignment with default parameters.

^b.^ Amino acid sequence alignment was performed using DNAMAN software using fast alignment with default parameters.

### Genomic structure and organization

**Fig 1:**
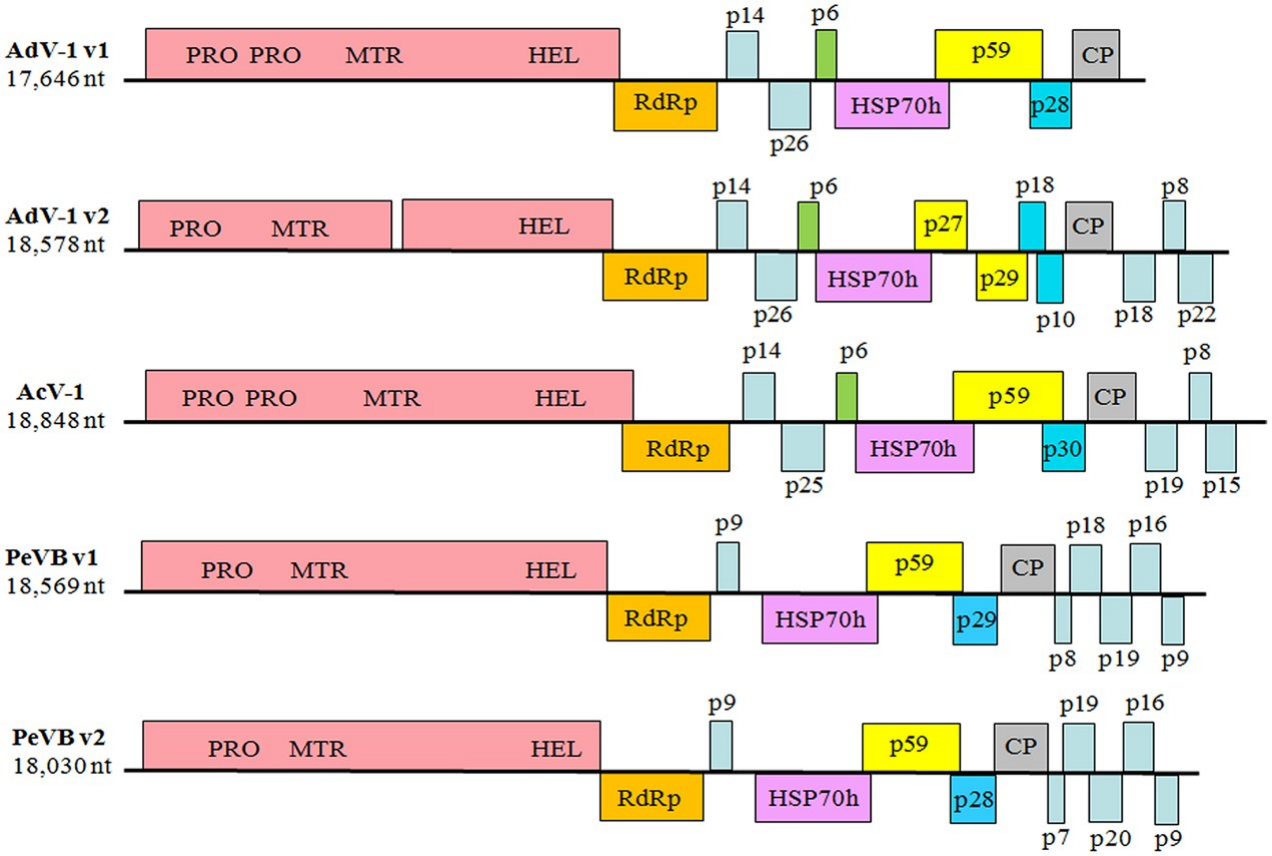
Schematic illustration of the structure of the genomes of AdV-1 variants and other relevant members of the family *Closteroviridae*. The names of proteins or motifs corresponding to acronyms are as follows: PRO (leader papain-like protease), MTR (methyltransferase), HEL (helicase), RdRp (RNA-dependent RNA polymerase), HSP70h (heat shock protein 70 homolog), CP(coat protein).

Sequence analysis conducted using Open Reading Frame Finder predicted that the two variants contained 9 and 15 ORFs, respectively, encoding for polypeptides ranging from 5.58 to 356.71 kDa in mass (S3 Table). Not only was the number of ORFs for the two variants different from relevant members of the family *Closteroviridae*, their genomic structure and organization were also somewhat different (Fig 1, S4 and S5 Tables).

The first ORF (ORF1a, nt positions 293–9850) of AdV-1 v1 consisted of 9,558 nt encoding a protein of 3,185 amino acids (aa). Similar to ORF1a of AcV-1, the conserved domains of two papain-like leader proteases, a methyltransferase and a helicase domain were also present in ORF1a of AdV-1 v1. However, it was divided into two smaller ORFs (ORF1a1 and ORF1a2) in AdV-1 v2. ORF1a1 (nt positions 122–5329) of AdV-1 v2 encoded a 1,735-aa protein containing a papain-like leader protease and a methyltransferase domain, while ORF1a2 (nt positions 5362–9693) encoded a 1,443-aa protein with a helicase domain. In the family *Closteroviridae*, the majority of members possess only one papain-like leader protease domain, although some have two, such as the *Blueberry virus* A (BVA, KF0 07212) [12] and *Citrus tristeza virus* (CTV, NC001661) [18]. Because CTV is classified as a C*losterovirus*, even though BVA remains unclassified, the number of papain-like leader protease domains present in ORF1a was therefore not a distinguishing characteristic. Two papain-like leader proteases may have evolved by gene duplication which function synergistically to enhance virus RNA amplification [19].

**Fig 2:**
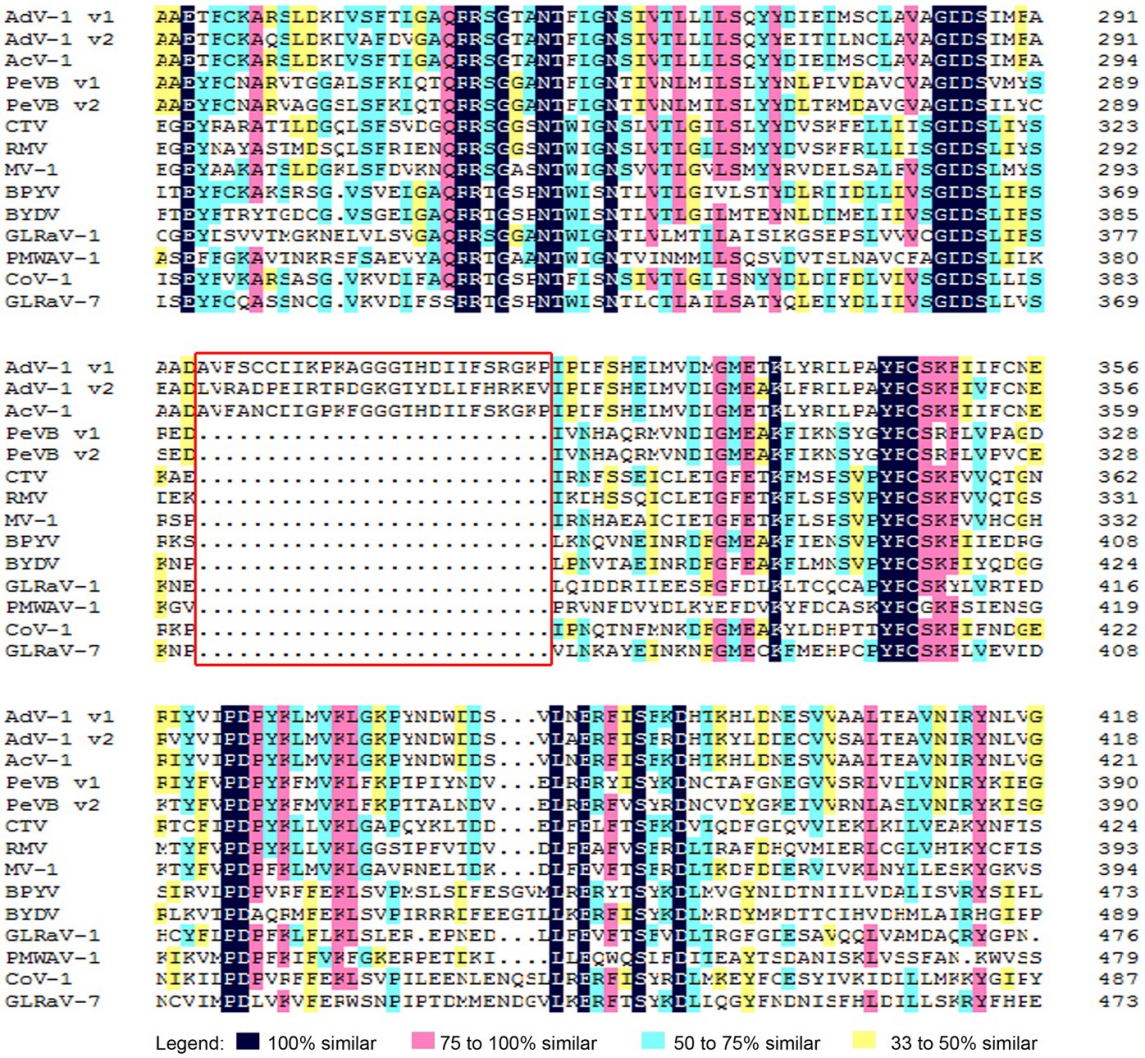
Multiple sequence alignments of RdRp proteins from AdV-1 variants and other species in the family *Closteroviridae*. Gaps (·) had been introduced to optimize alignment. The unique 26-aa inserts are boxed in red. The RdRp proteins of *Actinidia virus* 1 (KX857665), *Persimmon virus* B variant 1 (NC025967), *Persimmon virus* B variant 2 (AB923925), *Citrus tristeza virus* (KU883267), *Raspberry mottle virus* (NC008585), *Mint virus* 1 (NC006944), *Beet pseudo-yellows virus* (NC005209), *Bean yellow disorder virus* (NC010560), *Grapevine leafroll-associated virus* 1 (NC016509), *Pineapple mealybug wilt-associated virus* 1 (AF414119), *Cordyline virus* 1 (HM588723), and *Grapevine leafroll-associated virus* 7 (HE588185) are abbreviated as AcV-1, PeVB v1, PeVB v2, CTV, RMV, MV-1, BPYV, BYDV, GLRaV-1, PMWaV-1, CoV-1 and GLRaV-7, respectively.

The second ORF (ORF1b) of both AdV-1 v1 and v2 was 1,524 nt in length and encoded a 507-aa RNA dependent RNA polymerase (RdRp, S5 Table). Both RdRps expressed via a +1 ribosomal frameshift to fuse with ORF1a (Fig 1), a common feature of *Closteroviridae* and responsible for viral RNA replication [20, 21]. RdRp (nt positions 9822–11345) of AdV-1 v1 may be expressed via a +1 ribosomal frameshift by the sequence 5'-GUAAUAAGGUCACAAGCCGUUCAGGA***UAG***AA-3' (nt positions 9822–9852, the stop codon of ORF1a denoted in italic-bold), while RdRp (nt positions 9663–11188) of AdV-1 v2 may be expressed in a +1 ribosomal frameshift by the sequence 5'-GCGUCAUAAGGCCGCAGGCCGUUCAGGA***UAG***AA-3' (nt positions 9663–9695, the stop codon of ORF1a denoted in italic-bold). RdRp from AdV-1 v1 shared high amino acid identity (> 80%) with that of AdV-1 v2 and AcV-1, but had low amino acid similarity (< 38%) with the RdRps of other members of the family *Closteroviridae* (Table 2). As with AcV-1, a unique 26-aa insert was present in the RdRp motif (PF00978) of both AdV-1 variants (Fig 2), resulting in the addition of a coil on the surface of the structure without affecting the active sites.

There were three ORFs following ORF1b that coded for hypothetical proteins of unknown function. Their molecular masses were predicted to be 13.68, 25.56 and 5.58 kDa in AdV-1 v1, and 14.34, 25.83 and 5.67 kDa in AdV-1 v2, so these were termed p14, p26 and p6 in both AdV-1 v1 and v2 (Fig 1, S3 and S4 Tables). However, the similarity of the sequences of p14 in AdV-1 v1 in terms of both nucleotides and amino acids compared with AdV-1 v2 were low (< 57%), as they were in p26. Notably, p6 from AdV-1 v1 and v2 shared 86.27% amino acid identity with each other, and both harbored two transmembrane helices (S3 Fig), suggesting that they are transmembrane proteins.

ORF6 of AdV-1 v1 and v2 were both 1,755 nt in length and encoded heat shock protein 70 homolog (Hsp70h). Hsp70h (nt positions 12757–14511) of AdV-1 v1 had 88.87% amino acid identity with that of AdV-1 v2 (nt positions 12580–14334), and 93.15% with that of AcV-1, but only 33.28%, 35.18%, 24.32%, 23.72%, 24.83%, and 23.54% with those of PeVB v1, PeVB v2, *Mint virus* 1 (MV-1), *Bean yellow disorder virus* (BYDV), *Grapevine leafroll-associated virus* 1 (GLRaV-1), and *Cordyline virus* 1 (CoV-1), respectively (Table 2).

The seventh ORF (nt positions 14420–15943) of AdV-1 v1 encoded a hypothetical protein of 58.80 kDa (p59) that is 1,524 nt in length, which had high amino acid similarity (93.69%) with heat shock protein 90 homolog (Hsp90h; p59) of AcV-1. However, no specific Hsp90h domain was detected in p59 of AdV-1 v1 when searched using both the pfam and NCBI databases. Additionally, p59 of AdV-1 v1 shared < 30% amino acid identity with Hsp90h of other members of the family *Closteroviridae*, such as PeVB v1 and PeVB v2. In AdV-1 v2, ORF7 was divided into two smaller ORFs (ORF7a and ORF7b). ORF7a (nt positions 14243–14950) and ORF7b (nt positions 15011–15766) of AdV-1 v2 had 40.63% and 43.79% amino acid identity with p59 of AdV-1 v1, respectively.

ORF8 (nt positions 15780–16517) of AdV-1 v1 encoded a 245-aa, 28-kDa protein (p28), in which a motif of thaumatin-like family (PF00314) was present when searching in pfam database. A similar situation was also present in ORF8 of AcV-1 and ORF6 of the PeVB variants [17, 22]. This p28 protein shared amino acid identity of 77.36% with p30 of AcV-1, 23.35% with p29 of PeVB v1, 21.80% with p28 of PeVB v2, and 22.71% with p21 of olive leaf yellowing-associated virus (NC043417), another unassigned member of the family *Closteroviridae*. However, ORF8 was also divided into two smaller ORFs (ORF8a and ORF8b) in AdV-1 v2. Both ORF8a (nt positions 15603–16091) and ORF8b (nt positions 16076–16345) of AdV-1 v2 shared high sequence similarity with ORF8 of AdV-1 v1 and thaumatin-like proteins, although no thaumatin-like motif was found in either ORF8a or ORF8b.

ORF9 of AdV-1 v1 and v2 were both 732 nt in length and encoded a 243-aa protein that have the conserved domains of *Closterovirus* coat protein. Sequence identity analysis showed that CP in AdV-1 v1 (nt positions 16519–17250) had 88.07% amino acid sequence similarity with that of AdV-1 v2 (nt positions 16347–17078), 93.42% with CP of AcV-1, 21.25 to 23.08% with CP in PeVB variants, and < 21.00% with CP in other members of the family *Closteroviridae* (Table 2).

There were three short ORFs downstream of ORF9 in AdV-1 v2, encoding a 157-aa, a 70-aa, and a 191-aa hypothetical protein with unknown functions, respectively (Fig 1, S4 and S5 Tables). Amino acid sequence alignment showed that the three ORFs had 87.26%, 68.57%, and 55.50% similarity with those of AcV-1, respectively. It is a common feature of *Closteroviruses* that several distinctive ORFs present in the viral genome lack significant identity to known proteins [23]. Sequence alignment demonstrated that no proteins upstream or downstream of the *CP* genes in AdV-1 v1 and v2 had high sequence similarity with CP proteins. This suggests that both the genomes of AdV-1 v1 and v2 lack a minor CP, also observed in the genomes of AcV-1, the PeVB variants, BVA, *Mealybug wilt-associated virus* 1 (PMWaV-1), *etc*. [12, 22].

### Phylogenetic analysis

**Fig 3:**
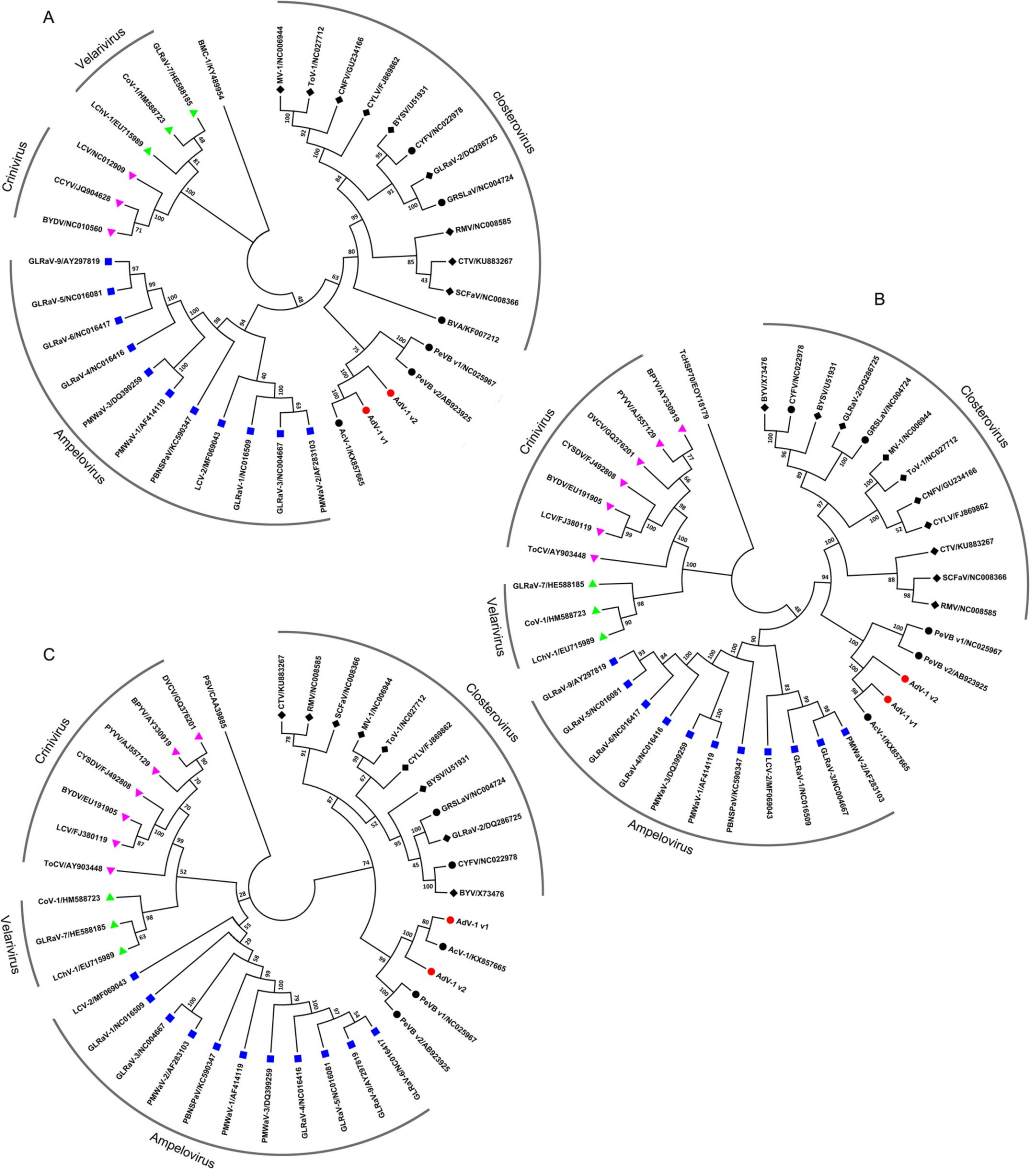
Phylogenetic trees of the amino acid sequences of RdRp (A), HSP70h (B), and CP (C) from representative members of the family *Closteroviridae*. *Bipolaris maydis chrysovirus* 1 RaRd (BMC-1 in A), *Theobroma cacao* HSP70 (TcHSP70 in B), and *Peanut stunt virus* CP (PSV in C) were used as outgroups. Bootstrap support values are displayed at nodes. Virus names and their abbreviations were *Actinidia virus* 1 (AcV-1), *Persimmon virus* B variant 1 and 2 (PeVB v1 and PeVB v2), *Blueberry viru*s A (BVA), *Grapevine rootstock stem lesion associated virus* (GRSLaV), *Carnation yellow fleck virus* (CYFV), *Mint virus* 1 (MV-1), *Carnation necrotic fleck virus*(CNFV), *Tobacco virus* 1 (ToV-1), *Ctrawberry chlorotic fleck associated virus* (SCFaV), *Beet yellow stunt virus* (BYSV), *Citrus tristeza virus* (CTV), *Raspberry mottle virus* (RMV), *Carrot yellow leaf virus* (CYLV), *Beet yellow virus* (BYV), *Plum bark necrosis stem pitting-associated virus* (PBNSPaV), *Little cherry virus* 2 (LCV-2), *Pineapple mealybug wilt-associated virus* 1, 2 and 3 (PMWaV-1, -2, and -3), *Grapevine leafroll-associated virus* 1, 2, 3, 4, 5, 6, 7, and 9 (GLRaV-1, -2, -3, -4, -5, -6, -7, and -9), *Little cherry virus* 1 (LChV-1), *Cordyline virus* 1 (CoV-1), *Bean yellow disorder virus* (BYDV), *Cucurbit chlorotic yellows virus* (CCYV), *Lettuce chlorosis virus* (LCV), *Tomato chlorosis virus* (ToCV), *Cucurbit yellow stunting disorder virus* (CYSDV), *Potato yellow vein virus* (PYVV), *Beet pseudo-yellows virus* (BPYV), and *Diodia vein chlorosis virus* (DVCV). Accession numbers of the sequences are listed after the slashes.

To investigate the phylogenetic relationships of the AdV-1 variants, three phylogenetic trees were constructed from aligned datasets of taxonomically relevant proteins (RdRp, HSP70h and CP) of the family *Closteroviridae* (Fig 3). The branching pattern of the phylogenetic trees demonstrated five distinct clusters (*Closterovirus*, *Ampelovirus*, *Velarivirus*, *Crinivirus* and unclassified group). The RdRp, HSP70h, and CP proteins encoded by AdV-1 variants were grouped with the unclassified virus variants PeVB v1 and PeVB v2, and AcV-1 in the same clade, consistent with their genomic organizations (Fig 1). A whole genome based phylogenetic tree was constructed (S4 Fig), which confirmed that the AdV-1 variants fell into distinct clades apart from the four existing genera under *Closteroviridae*. Notably, like PeVB v1 and PeVB v2, AdV-1 v1, v2 and AcV-1 were found on a species-specific cluster in the unclassified group. This placement confirmed that AdV-1 v1 and v2 were two variants of a novel member of the family *Closteroviridae*, homologous to AcV-1.

## Discussion

High-throughput sequencing is not only used to analyze the expression levels of known genes from multiple organisms, it can also be used to detect new genes and new variants [24–26]. Compared with Sanger sequencing, transcriptome sequencing using next-generation sequencing (NGS) technology provides greater throughput and broader coverage [27]. Different strategies for library construction can be adopted in NGS depending on the specific research objectives. Previously, NGS technology was principally used to analyze nucleotide sequences and expression levels of mRNAs because the sequencing library was constructed from RNAs enriched with polyA. In recent years, a new strategy of sequencing library construction was developed by the removal of rRNAs so that long non-coding RNAs could be detected [28, 29], facilitating the simultaneous detection of viral genomic sequences contained in the same samples. In the present study, two new viral genomes were obtained simultaneously from the same kiwifruit sample when sequencing for long non-coding RNAs. Overlapping RT-PCR and RACE-PCR confirmed the reliability of the two new viral RNA sequences (AdV-1 v1 and v2). In addition, AdV-1 v1 contained nine ORFs with a 292-nt 5'-UTR and a 396-nt 3'-UTR, while AdV-1 v2 contained 15 ORFs with a 121-nt 5'-UTR and a 156-nt 3'-UTR (Fig 1, S4 Table), different from those of AcV-1, PeVB v1, and PeVB v2 [17, 22].

Although the genomes of AdV-1 v1 and v2 contained different numbers of ORFs, homologous ORFs shared high sequence identity at both nucleotide and amino acid level. Furthermore, the amino acid divergence values of 6.47 to 19.53% for RdRp, HSP70h and CP from AdV-1 v1, v2, and AcV-1 were within the normal range for variants, in accordance with previously reported threshold values [11]. Phylogenetic analysis also revealed that AdV-1 v1 and v2 had a relationship closer to that of AcV-1 than other members of the family *Closteroviridae*. Therefore, we hypothesized that AdV-1 v1 and v2 represent two variants of the family *Closteroviridae*, homologous to AcV-1. In addition, similar to AcV-1 and PeVB variants, results of BLAST sequence and alignment indicated that RdRp, HSP70h and CP in the AdV-1 variants displayed low sequence identity (< 35%) with those of other members of the four identified genera (*Closterovirus*, *Ampelovirus*, *Velarivirus*, *Crinivirus*; Table 2). Interestingly, the ORF containing a thaumatin-like motif (PF00314) or sharing high sequence identity with thaumatin-like proteins was present in AdV-1 variants, AcV-1, and PeVB variants, but absent from members of the four identified genera of *Closteroviridae*. Together with their phylogenetic relationships (Fig 3, S4 Fig) and genomic organization (Fig 1), we speculate that the AdV-1 variants, AcV-1 and PeVB variants represent a novel subfamily of *Closteroviridae*, different from the four known genera.

This is the first report of the genome sequence of *Closteroviridae* viruses from kiwifruits in China. Some *Closteroviridae* viruses are devastating to their host plants, such as CTV in *Citrus* [30]. Recently, leaves from different varieties of *Actinidia chinensis* (cv.s Hongyang, Donghong, Honghua, Jinguo, and Jinyan) collected in the Sichuan Province in China, were used to perform RT-PCR using primers designed from the CP gene of AcV-1, the results indicating that these isolates shared 89.8–90.2% nucleotide sequence identity and 94.7–95.9% amino acid sequence identity with the CP of AcV-1 [31]. This implies that variants exist, consistent with the results of the present study. Furthermore, this virus was detected by amplification of the CP gene of AcV-1 from both symptomatic (*e*.*g*. leaf mottle, chlorosis, or malformation) and asymptomatic leaves in Sichuan Province [32]. Similarly, the genomic sequences of AdV-1 variants in the current study were isolated from asymptomatic material from *A*. *chinensis* var. *deliciosa* ‘Miliang-1’ in Fujian Province. Hitherto, this virus has only been found in Fujian in the southeast of China and Sichuan Province in the southwest, and it is unknown whether it exists elsewhere because of the lack of prior detection. Since *Actinidia* plants in the field are often co-infected with several viruses, the precise role of this virus as a causal agent remains unknown. Therefore, future studies should focus on its detailed distribution and pathogenesis to avoid a large-scale disease outbreak and economic loss.

## Supporting information

S1 Appendix Genome sequences of *Actinidia deliciosa virus* 1. (DOC)

S1 Fig Sequence alignment of AdV-1 v1 and AcV-1. (EMF)

S2 Fig Sequence alignment of AdV-1 v2 and AcV-1. (EMF)

S3 Fig Transmembrane helices of p6 from AdV-1 v1 and v2. (JPG)

S4 Fig Whole genome based phylogenetic tree. (JPG)

S1 Table Oligonucleotide primers used for RACE-PCR of *Actinidia deliciosa virus* 1 variants. (DOC)

S2 Table Sequence identities of *Actinidia deliciosa virus* 1 variant 1 and 2 with other members of the family *Closteroviridae* analyzed by Clustal X. (DOC)

S3 Table Molecular weights (kDa) of proteins encoded by the ORFs of the AdV-1 variants and AcV-1. (DOC)

S4 Table Nucleotide positions in the nucleotide sequences of the AdV-1 variants. (XLSX)

S5 Table Lengths (nt/aa) of nucleotide sequences and protein sequences of the ORFs of AdV-1 variants and AcV-1. (DOC)
