# Supplementary material for: Characterization and complete genome sequences of two novel variants of the family Closteroviridae from Chinese kiwifruit
Source: PLoS One. 2020 Nov 23;15(11):e0242362. doi: 10.1371/journal.pone.0242362 (PMC7682855; doi:10.1371/journal.pone.0242362)
Supplement: S1 Appendix — (DOC) [file pone.0242362.s001.doc]

**S1 Appendix. Genome sequences of *Actinidia deliciosa virus* 1**

**>*Actinidia deliciosa virus* 1 variant 1 (AdV-1 v1) genomic RNA, complete genome**

ATTATTTCGCAGATTACTTTTGCTGTTGTATAAGGTTGATTCTTATATTGCAGCACATATCGCACTAAGTATCTTGGTGCTTTATTATAATTTGCAAACAGTTCCCGCTCTTTCTCCGAGCTCTCTGCTTTCTTGATCGTGTGCTCTGGAGTGAGAACCTCTCGCACACAGTTCCCTCTCCTCCGTGCAGCTCGCATATTGTGATTGCCACTCTTAGGCGTTGCTGTGACTGCTTTTAAAGTTCCATAAATTAACCACAACTAAACATCCGTCAAACTCCTCCACTTCCCGCATGGCTCCCCCGCGCTTTTTAAGAGCGTCGCCGGGAAGGGCCAAGGCCTCTGTGGCCTCCAGGCTCAACATCAAGGGAGAGTGCTCCAAAACTCTCCCGAAAGGATGGGGTGCTGATGCCTCATTCACTGTTGCGCGCTTCTTTGGCGCGGAAGACAGGGAGATCACTTTCTCCCGAGGATGCTCTCGCATCCTCGATGCGTTTGGTCTGAACACCCCCTCTGCGGTGTTTTACGGGCCTGAGCCCGCTTCCCCTGCTTTCCTGAAGGCTCGCACCAATCGCGAGCTATATAGCGGGGTCCAACAGCCTTTCAATTCCCGGAAGGCTGTCCGGCAGGCTGCAGTTGCAGCATCTCTGTTCGAGGCCCGCAAGAAGCGCGCCTCCAAGGTTCATCTCCCGGTTATCGGGAAGAAAAGGAAAGTGGGGCCTGCTTTTAAGGCACCAAAGAAGGAAGTCGTCGAGCAGACGATGGTTCCTGTGGCGGCACCGACTGTGCCGGTGGTGGTTCCGACCACCTTTCCAGTCGCCTATGTGGCGAAAGGGGCAACACGATTGCCCGAAGGTACCCCAGTCTTCCTGGCCAGGAAACCTGCTGCCAGGACTCTGGGCTTCAATATGGCCCGTAACCAACGGGAGTTCATCCGTCTATCTTTGGACGGATGGTCTGTTACTCTTGACACCGAATCGGGTGTCGTGACTGACCAGGCTTTGTTGGCCCTGGTCAAAGGGTACCACTACGAGGCTTTCTTGCCTCTGAAACTATTGGCCAAATATGGGGCCAAGCTGACCCCGACCTTGGACTACGGTTGGGTCAGATACGACCGCAGCGGTCGTATAAGCAGGTTGTTAAACCTCCCGTATATGTGGGAAGTTTTACAACTTCTTAAAAGAGGGGAGTGCGGGCCTAAACTGCGGGCCTATATAGAGTCTTTCCAAGATAACAGGGGTTACTGTTATCTTAAACTTTTCAGGATGGCCAATATAGCCATCGGCAGATCGGCCAGAAGGGTTGGCACGGTGTGCCATTTATTAGGGCCTTTCCCGAGCACTCGAGAGGTGCAGACGGTTCTGTACCGTCGTTACGCGCGTATACCCGACTTCATTGTGGGGTACAAGTCGCAGGGAAAAAGGGGGCACATGACTACTACCCCCACGACCAGAGTATCCGCTCCCCCAGATTTATACTGGGGGGCTGACTGTCTGATGTTCGGTTCTATACCGGCGTCAGTCCCGGTAGAGGAGGTGATAGCGCCTACACCATTGAAGGTGGGGCGCCCTGTAAGTCCAGCGGGGACTTCCACGGAGCCGCGCATCGTCTTCGGCAGTTTCGGAACGGCCGCGCCGAGTCCGCAAAAGCCCCCGATTGCGATGTCAAAATCAGCAATCCGTAGATTGCGCCGAAAGAATGCGGTACTTCGCGCTGGGGCTCAGAATATCGATCGAAAGATCGATACAGTGGTAGTTTCCCCTCCCCCTGCTGAGGTTCCTATGAGAGTAGAGACCCCAGCTCCTGTGGAGAAACAGAAAATTGCGCCTCCCGCCACTACCTTCATCAAACCGGTTTTTAGAGACGTGAAGCGATGTGGTGGTCTACTTAGAAGCTGGTTTGGTAAAGTCAACCCACACGAAAGGGTAAAAGACAGGTTGGCTTCTCTGTTGTCCGCTGATGGTTCTGGCTATAATTATAATGGGGGTAGTCATAGACCCGATAAGAGGAGCAAGATTCTTTTAGCCGAACTATCATCTATCTTGGAGATGGATCTCGGTTGGGTGAAGCACGCTCTAGTGCAAAAATACAGACCAGGTTCGAAGATTGGAGCTCACAAAGATAACGAACCATGTTATCGGCCTCTGTGTAACTTCAGGCTGGTGACGATAAATGTCTTTGGGGAGGCTCTGTTTAGCTTGAGCAGAGGGGCGGAACGTTACAACATAGGTCTGGACGGACCTTGTATGTTCGAGATGGACCCAAGTGTCAATTTCCACTTCGACCATAGTGTCGAAGTAGGGAGATTTTTCCGAGGTTCTATCACCCTCAGAGGTCACAAGAATTCGCATGTCCTAGATCGATCGCAACCGTCAACCGGTGCTAAAAGGAATGTCGAATCGGAGGTCGTAGCCAAGAGGTTCGCCGTTTCTTCGGAGAAGACCAGACCCTCGGCAGCTGAGGGTCCTAAGCCTGTGACCTGTCCACCTGCGGTAGAGCGGATTAGGTCGATATCACCTCCAGTTAAGACGACCGGTTCTAAAGAGAACCAAAAACCTGTTGTTCGAAGGCAGGAAGAAATTAGAATCGATCATCAATCCTATTTGGATGCTCTGACCCGGTGTAATTCAGTGGGTGACATTATAGACAAAGCGAAATATCCAGATTACAGAGCGGTATGTCAAAAAAGCAGTTATGGTTTGGTATATATATACCATAAGGATGTGTTGGTGAAGAAGGGTATCTTTAGAAGATACTACGATATTAAAGCGCTCCGGCAGTTGAATCAAATTACAGATAATCTGAGAGCATATCTAAGCAGCTTCAGAGATAGCAGCGGATACTGTTATCTCGTTTATATTAGAGCGGTGGCCATGTATTTCGGTCGAGCGGAGACGGAATGTGCAGTCGCAGTTAGAGCTTTAGGACCTTGGCCTAAGGCTGGAGATCTACTGTCTTACATACACAAGAGGTATGGGACCTGCCCTCCTATTAAAGTCGGTTACAGACATGTAGGAGGTGCTGCAGTACACGCTAACCTGACGCCAGTCTTCCTTTTGGCTAATATGCGAAAAGGGTTACGGGTCGGAGGTGAGAAGACCTGTCGTATCGATTCTTCATCTATAATGGTCGGGACAGTGGAGTGCCGTATTTCTCGTTATGAGCCTATCACTCCTCTTTCAGGTTCTATTAGAGTCTCAAGTGAGCGACAGGAGTGCTCTAAAAGTCCGATTAGGATCACTAATGTGGGCCTAGACGTCAGTTTGGCGCGGGTGACGGCTGTTAAAAGCTTTAGAGGAAGGCAGTATAGATGCAAACGGGCCGAGCGTGACGTCGGTTCAGTGGATAGCGACCTTACTTTAGCTGCAGAATCGCAGGCTAGTTGCAGTGTGGCCGATTCCTCCCTCCGTGGTTGCGACTCGCGTAAGGAGTACAGACATCTTGTGAAGGGGCTTGTGGATAGAATCCTGGATTTTAAACAGGACGAATCAACGTTGAACACCCCCTTTTACGAGGGATTCAACGTTGTATGCGTTAAAACCAGGCCGGGTTTGGTGAGGATACTATACAAGAATGTTCTCGTTAAAACCATAATAGCCAACAGGTATTGGGATATACAATATCTTAGGTCTTACGGCCGCGTTTCCAGAAGTTTGAAGAATTACTTGTATAGATACGGTGATGGAGAGGGGTATTGTTATTTAAGAATACTGAGGTTGTGTTGTATATATTTTTCTAAACCTATGGGTTACGTACGGACAGCCAAAGCTGAATTAGGTAGCTGGCCTAGCTCACTCGCCGTGAAATGTTTTATCAGGAAAACATTTCCGAGGATACCCCCGGTATACGTATCGCTATCTAGAGGTAGATACGCGCATGTAGGGTTGCTACCTAGAGTCTGTTTAGAAAACATCCCGAATTTCTTAAAGCTTGGGGGGCACGTCGAGTCGGATACGTCGATGAGACGTATGACAGAAGTAAATAGGTTGAATGCGCAGGTGGAACGGGCACAATTAAAAGATTCTGCTTTACTGCGAGCGGTGGAGAGTACGCTCATTGAAGAGCACCGTATAGAGAAGCAGATGCAGAATTCGAAACCGGTACTGAATGTTAATGTCGCTTTGAATGATAGTCAGCAATTGGCGCTGACAAGGAACTTCCCCGAAATGAGGCTAAAATTCGTGCCTTCAGTACACTCTCTTCACCCGATGAGCTCTGCAGTGAGGATGTGCTTTAATGCGCTATATTCACAAAAGTTGGGAAAGAAGAAGTACATAGATATCGGAGGGGATTTGAAATATCATGTTATGAAGGGAAATGATGTCCACATTTGTAACCCGATTTTAGATCCTAAAGACGGGGTTAGATACGTTAATAGGGTGTGCGAATGGAATCTGGCGAAGGCGCACGATTTGACCAGTATGGTGGCAGGAAGTAAAAAAGTTTCATGTTGTTACACTCCCGCCCAGTATTGTGACGTATCTTGCAACACTGCTGTGGCGGTGGAAGTGTACGATATAAGTATGATCGAAATGGCGACTATTATGGCTAAGCGAACTATAGATAGAGTATACTTGACCATGTTGATTCCTGGGGAGTTGTTTGACGCTAACTCGGAAACGATATGTGTACCAGAACATGATATAGCCATATCTCAGGAGGGTGATAACTTGATATATAATATGCCTGCCGGTCAGAGCTATTGCCACGATAGATCTAGTGTTCTATCGTACATCCAAAATCCGTATTTGTTGCATGAGAATCAACTTTTCCATTCCGAGATGGTTGGTCATAGGTGTGGTGTATGTGAATTTAGGGTGACCAGAGTGCCAGTATATCCGGCCATTGATACGATAGTTCACGTAACGGTTCCTAGAGCCACATCAGGTTTAGTTGAGTTGCATCTGCCGAATATAGATAAGTTTTCGGACGTACTAGATTTCGATAATGTCACGTCTGTTATGGTGGATTATGACTTCTTTACTAGGGCTCTGACCCATATCATTAACGTGTGTACTAACGTTTCCGAGAAGACTTTCGAGTATACTATGACATGGCTGAGAAATAATTCAGCCAGAGTTGTCATATCGGGTCGTATCATACACACCAACGTTAAGTTGGCGCCGGAACATATAGGAAGAGTAGCTGCTTTGCTGCTCACTGCTGGAGTAAAAACTCGGTGGGAAAGCGGCAGATATGCCAGACGATTATACGGAGCGGTAGGTCAAGAAACGTTATGGGAGTCCATAAAAACTACTATACACGAATCTACATCAACGGTTAAGGCTGCCGCCTACGAGATGGTTAAGAAAATTCTGTCCAGCTCTTTTCCTTTTTTGGGAGATTTGCAGAGCAAGTCAATTGACGATTTTTTCACTGTTTTAGGCGAATCCGTAACGATCACGCGAGCTGTGAATTTCCCGTGTAGCGGTGGTTATATTCAAGGTGAATCTCGCTATATTGATAACATGGTGAATACTCTCCTAGCGGAATCTGTGGAGAACAGTGCTAGGTCGGAGATCGCTGAAGCGATGTCTGAAGTAATTAATAATGACGGTAAGCAAGATGGTGATAATAAGTACGTCGCACCGGGTTATAGGTCAGGGAAAAGCTCCTGTATTGTACGAGAGAAGATAAAAGCGGTCACTGGTACCTTGAATCGTTGCGATGGTTCTAAACCCGGCCAAGGTGCGGGGCTTCGTGATAGTGGATCGCCTTTATTTTTAATGATATTGCGAGTTGTTGAAGGATATGTAAGCTTAAGTGTGAAAAAATTTAGGGACATTCTCCTGCGTATAGTATCTCCCTTCCCAAGGATTAGGACCGTTTGGATCCCAATTTTGAAACTTTGGGAAGGTTTATTCTCTGGTGATGCGGATGTGTGGGTCACTTATGGTGCTACAGTAGTTTATTCCGTAATACGCTCAATTGTGTACTTATTTTTAGGCCACTCAACTTTTGGTGTGTGTTTGGGTCTGATAGCAGTTTTAGCAACCCCGATTCCACCGCTTTTTATTAAAGATAGGGATAACTTATCGGCTGACATTTTGTTCGAGGCTCTGAAAGGGGCTTATTTTTCAGTACCTCTAACCGGCAACAAGTGGTTGAACAGAGTACTTTCGGTGTTGGAGAGTGTAGGTTATTTTAAATCTCTCGTGAGGAGGATCATAGCGGTTGTTTTTGAAGAAAGCACTGCAGCGTCTGTGGTTATGTTAGTTATACCACCGGAAGAGAACATTTGGGCTATTAAAAATTTGGTCAAGAAGGCTTATGATTGGGCCTACGATCAAATCTGTACTACTCTTTACGCTTTGTTAAGTGCTGTACCACATTCAGCGAAAGCTGCAGTTGCTAACACCATCGGGGACGTAGCAGGCGGTGTGTCGTCTGTTCTCGCGACATCGGTAGCTAAAGTTTTGGACTGGTTCTCTAACCAACGACAGAACCCACCAGGGTTAGATCAGGATGGTGGTACTGAGGAGTTCTTTAGTATGGTGGATGACGTCGAGTCGATTGATGACTTTTTATCCGAAACTCCCGGTCTTAGAGGAGGAGGTGTTTTTAATAGGAATTTCTTAGCCTCTTTGATTAAGAAATTTTTAGATATGGGCCGGACGTTAGTTGACAGTGTGATGAGCTCTATAAATTACATTAAATGTAAACTCTATCCTGGATCGTTGGGTAGGACAAAGAAAGGGAGTGAATTACTGGACGAATTGTTCAGTGAAAGAGATTGTGAAGATGAAGAATATGACGCGGCGGTCTTATACCTGAACGAATTCCTTAATACAGATTTTTCAGACGCTCCAGGTAACTTTGGTGGGGCTGTAGACTGTAAGTCATTCGTAATGTCTGTTTATAGGTACTTCAAATCGTTCAAATTTAGTAGTGTCGTAGCAATGTGTCAAGCAATCCTTGCTTTTTTTGTTATGACTAAACGTCTCTGTATGGTAAGGTACAGAGTGCTTATGGCTGAAGTCAAAGCACGCTTAGCTAATTACAAGAGGAACAATCCTGCAATGGCACTAGCTCGATTAACAGAAGTTATGGATGACGATGAAGATCGGTGCTACAGAGTCTCTGAGGATTTATTTGATGCGGACGAGCAAGTCAGAAATTTGTGTAGAAATAGCAAACCTCAGTATTTCAAAAGGATGGACGTGTACAGAGTTCCTACTGTTATTGATTACGAGGATAAAAACGCTCTGATCGGAAACTTGGTTGCGCCGGCTATTGATTTTTTGATGGTGCACGATAGTTCAGACGTGAGCGCAATAGGTTTGGATTTCAATCTTATTAAGAAAGTGCTCGGTTGGACGATGACTAAGGATTTAGTAAGTAGGATTCCATTAGCCAGGAGATGTTATCCAAACTCGATATTGATGATTCGGAAAGGCGGTTATGCTATTTTTAGCGCCAACGGAAGACCTATAGTCAGCAATTGTTTGAATCTGCATCTCACTCCTGAAAAGTTCGATGTTATCTTCATGAGATTGTCTGGCGGTCTTATGGGGGGCGGTGTTGCTTCTTGGAGTCTAGTGGTTTTATTCAGGTATCTTCTTGATTTGCTGGAAGAACATAGTGTTATCAGCTGTCACGTAAACGTAGCTTGCAAGGTTGTTGCATGCGCCACTTCATCCGTCTGTAGATGGGTTACGATGGCTGACTGGATCTTGAAAAGAATCTGTGATTGGTATACTCATGGGAGACATCGCCAGAACGAGATGGAGATAAAACCGCTGACCAAAGTTGAAGGAAAATCCGTCACTATATCTAGTGAACTTAAGAAAGTATACTCAGATACAGTGGAGGTGAAGGCTGAACACATGGATGAATTGGTAAATGAATTGGTCTGTCGATCTTCGGATGATGGAAGCGGTACTACAGATCCCGAGATCAGTTCCGAGGACAGTGAAGGAAATTCTTATAATAAAAAATTCTTTAACAAAAAGGTAAATGACGAAACTGATCGCTTAAGACGTGAAAATCCTGTTAAAGGGAATGAAGTCAGGGGTAGGAAGAAGACGAGTAAAAAGGGTAATAAAGTTCCTACCAAGTCCTGTGAAGAACGGAACGACTCTGACGAGGCCGAAGGCAGGTTTAAGGTATTAAACCTTCGCAATGAGGAGAAAGAACGGTTCGGTAAATTGTATGGTAGTAGGAGGTACCAGCTAGCCGAAATTATAAGGAATCTAGACCTGACTTGTCCTCCGGTTTTTACACACACCGATGACTTGGCCGTAAATGCTATGAACGAATTTGTGTTCTTGCATTTAATGGACGTGATGAATATGTTGAATAGTATGAAGATAGCATCATCGCTCTTGGTCAACGATAAGAGGAACCCTGATCTTCTTAGATGTGATATGGTGGACCCTAAGATAATTGTTCTAGACACCACCACCGACTTGTTGTGGAATACTGTTACAGCCACAGTTCACTTGAGAGATACTCAGCACAGGTTTTGTTATGACCCTAAGAGTAATTCGATAGTATCGCTTGGAGCGTATAGAGTTCACTCCTGTTCGAGGTATATAGTATTACATCAAGATCTTGAGATCTTCTATGCCAACCTAGTCCTGAGTAGATTTGTGGTGAACGAAAAGGTTGTAAAAACTCACTACTTAGACGGCCTCGCAGTGGTAGAGACTCCTCCTGGCGGTGGGAAGACTACCCAATTAGTTGCGTTATTTTTCAACTTATGGATGAGAGGCGTCTTTGTCAGAGTCGTAACAGCAAACAAAAATTCTGCTGAAGAAATAAGGCGTAAGGCGAGTGCCTTAGCCGTACATTTTAAAGTTGTCGAGGAGAGGTATATTACCAAGCTGCGTCAACTTTTAGATGACATGGTGAGGACTGTCGATTCCACAATAATAAATGTGGTATCTGCTAAGACCCAAGTTCTGTTGGTAGATGAGATTTTCCTGATGCATCTTGGTCAGTTGGTACTGAACATTGAAATTTTAAAACCTCAGTATGTTATAGGTTATGGTGATTCGAGGCAGATATCTTATATACCCAGAACCGATCTATTTTGCCCTATGTACTACAGGGTCAAGGATGTTATAGAGAGTGGAAGAGTTATCTACAGGAGTGAATCATATAGGTGTCCCAAGGACGTATGTTACCTGTTGTCTGAATTGTATGGCAGATCTATTGAAGCCAGAGTGAATAACAAAACTGATACTATGTCTGTTGCTTCAATAAACTCTATAGAGGACGTGCCCGTAGTCGAGGGTGCTAAATACTTAACGTACACTCAGGGTGAGAAACTCGAGTTAATAGCTACACTTCGACGGAAAGGCAGGAAGCCGTCGGTATACCTAGACCCTCAGACGGTGCATGAAGCTCAAGGCAACACATATAAGAAAGTGATTTTGGTGAGATCGAAGCCTCAGGATGATAGTGTGTTCAGCTCAGTCGACCATCACATCGTCGCTTTATCGAGGCACACAGACTCACTTGTATATTATTGTATTTCATCGAAGTATAACGATGATACAGCGTCAAAAATAGAGAGGTCCAAAGTACTGTCGTCTCTAAATATGAATGAAATCAATGAACAACCTATATTTGGTGCAGAGTACGAATACTCTGGAGGTAACCCCGAAGCAAGTTGCAGCAGAGCAGGTGCTATGGGGTGGCAGGCTATCGTTAGTTTTTTGGATGAAGTCGTGCCCGGGTCTACGGTTCTGACACTAAGTGATATCTCGGAAGCATTATCTACATCGGAATTCGAGAGTTGTGTGGATAAAATTAGAATCGGTGAGAACATGACCGTCGGAAAACAGCCTTTGCATTCGAACTGTCAGCGTGTTTGGCGTAATAAGGTCACAAGCCGTTCAGGATAGAAAACCTACCGTTCAAGAGAACATTTATAGTTATGAAGCTAGAAATTTCGTCGCCTTAACGTTAGACCGGCATTTGGACCCAGACTTGTTCAGAGATCATGCTGTGAACAAGTTCTTTAATAAGTGTGTGAACTCAGAAGTGTTACGGGGTCTGAATGAACAACCTATAGTCACAAACCATCTACATTTCCAGGAATGGCTGAGAAAGAGAGATGGTTCTGCTTTGGCAAAGTTGGACAATGAGGTTGGTTATATTACTACTTGGCGAGATTACATGTGTCTCTTTAAGCTTATGGTTAAGAAAGAGGCTAAAGTTAAACTGGACGCCTCTTCTTTGACGAAACATAACCCTGCCCAGAATATCATCTACCATATCAAGTTCATAAATGCAGTATTCAGCTCCATCTTCGCACAACTATCAGAAAGGTTGAGAGTTGTGCTCAAAAGAAACATCATCTTGTATACTAGTATGTCGGTGGATAGTTTCGCCGATCGTTTGTACGATGTCTTGGGAGGCACCAATGTGTATAACACTGTAGAGATGGATTTTTCAAAGTTTGATAAATCACAAGACGTCTACGTCAAAGCTTGCGAAATGGAAATTTATAGAAGATTGGGTATGTCAGAAGATATGCTGGATTTATGGTGTGCTGCGGAGACTTTTTGCAAAGCCAGATCCCTCGATAAAGATGTCTCTTTCACTCTAGGGGCGCAAAGGAGGTCGGGTACCGCCAACACTTTTTTGGGCAACAGCATAGTTACGCTGTTACTTCTTTCCCAATATTATGATATTGAGGATATGAGTTGTTTAGCCGTTGCAGGCGACGATTCTATTATGTTTGCCGCTGCTGACGCCGTGTTTTCTTGTTGTGATATCAAACCTAAGGCCGGCGGGGGCACGCACGATATCATTTTTTCAAGAGGAAAACCTATACCCGACTTTAGTCATGAGTTGATGGTCGATATGGGGATGGAGACGAAGTTGTATAGAGACCTCCCTGCATACTTCTGTTCCAAGTTTATTATTTTTTGCAATGAGCGTATATATGTTATACCGGATCCGTATAAATTAATGGTAAAGCTTGGGAAACCATACAATGATTGGGACGACTCGGTCTTAAACGAGAGATTTATTTCGTTTAAGGATCATACTAAACACTTAGATAACGAGAGTGTCGTGGCTGCTTTGACAGAAGCTGTCAACATCAGATACAATTTGGTAGGCTACCACACGTATGCAGCGATCAGCGCATTACACTGTGTGTCGGCAAACAAGAAGAGGTTTTTCGAGCTGTATCCTTTCAAGCACGGTTTCATTACCAGAACGGTAAGAAAAGTAGGTAGAATTATTTCGAAGTTCGCTCATTACATCAGGTCTAAAGGGTTTATCATCATTGACCAACGCGATCAAGGTGATGCATACACTTTCGATTACGCTGTCCGGGATATCCACAAAGCTAGGAATAATCCGGATGTTTTTGGGAAACCTATTTAGAGTGGGACGAATACACTGTTGCCTCGAAGAGCCGCTCGATCGTCATTTAACGATCTGACGATGAGTCTCATTATTTAATGGAGAAATGATTTAATTGCAGTTTAATAACGATAAACACGATTACTACTGTACGTGATATAGGACGGTACACAGCAATTAGTGAAGGATAGTTAAGATGGGTGCCCTTTTTACTGTATACGAATCAGAGCAGTATGTAGTGCGCGACAATCGCGGTCGACGCCCATATTGGGAATCGGGCTATCATAACAATACGTATTGTGACGATTGTGGTCCTAGCAGACCCTATGATCATTCAATGAGCTCTCTGGTTGATTATTACAATCGCTCGGACGTGCAACAACATCTCGTACGTCGTGGAAGAATACTTTCGGAAAATTTAATCGAAAGAGATAATTTATATGATGCTGAGATCCGTAAGCGTAACGAATGCTACGCTAGACATAAGAGAAGGTCGAGGAAAAGGTTTAGTCTGTACTCTTTGTTGTATCGATGATAGGTAGCGTAGGTTTATATTTAATTATTAGGTCAGTGCATTAGATTACTAAAACATGAATTGTTTCTTAAGGTGTTCCCGAGTTTACGATAACTGGGTGGTGACGGTTAATGCAATCCTTTTGGGTCTTAAGATGTACTGTTATCTGAAAAGTCGTGTAACCGTACTATTGGTACTTGTGATTGACAGTTGCATGGATATGATCTTCCAAGCCTTGAACTTGTTGGGCAATGTATTCAAGACTAAGATATTTTTGATCGTAGCATATGCGTCTGTCATACTTTCACAAATTTATCCTTTACACCTAGTATCCTTGTCATTGCTTAAATCTCATGATTACGAAAATACAAATACGTATCAAATTGTCGTTATGACTCTATCTGTGTTAGTTTTCCAATTAGTCGTGAAGATGTTTATGTATATAGTTGGTAAGGTGAATTTCAATACTATAGGGAATGACGCGTTAATAGCGGATCAGTTATACGATGTATTAACTACTTTTATATCGATAGTTTTCTTCTGTATGATTATACAATTTTACTCACCTATTTCTAATTTATTGGACTACTGGGGAACTGTTATATTAGTTCTAGTGTCTCTAACCTTTTGGTTGAATAACTGGTATAGTAGGGATGATTGCACTACCGTCATTAGACCGACAGATGTTCTAAGTTCGAATTCTTCTCCACCCGTAGATGGTCGTGACAATTTACCAATCGTCGTTAACTAGTGGATAAGTTGTTGGATGGATGAACTACTACAAGTGGCTTTTTCATTACTTTTCCTTTTAGGATTTTGTTTATTATTGTGCACTTGTCTAGTAGGAAGCTTTTCCGTTTATAAGAAAGTAAGTGACCCAGACGCTGTGACGGAAAACGTCGGCAGGGTGGGCAGGTTATGACGATCATAGGTATTGATTACGGGACTACTTTTTCGACGTGCAGTATAGTCACGTCTACTTCAGTTTTTATACTCCAACACAATGATTCAGAGTACATACCGAGCCTTATTGCTATAAGTACCAAAACAGGTGCTGTTACTATAGGAGTTGATGTTGTCGGCAAAGAGTTGGATACCTCATATAGTTGTTATAAGGATATGAAAAGGTGGGTAGGAATTGATACCTCTTCTTATGCCGAAAGAGAGCTCAAACTTAAGCCTACCTACGATACTAAACCTCATGAAGATATGTTCGGTTTCGAACTTGGTGCTTATAATGTTAAAGGAAGATTAATGCCTATTAGGACTTTAATATCACTCTACATAAAGGCTCTAGTTAAATTGTTTGAAATTCGCTGTTCTGTTGTGTGTAGTGGTTTGGTATTGTCGGTACCTTCTCAGTACACCACATCACAAAGATCTTTCATGGTAGCGTTAGCTAGCGCGATAGGAATTAAGATAGTTCACATTATGAACGAACCCTCGGCAGCATTATTCGCGTCGGTTAGTAGTATACCCAATAAGGTGGCTAGTGAGTATTATATAGTTTACGATTTCGGCGGTGGAACCTTTGATGTTTCTATAGTAGGTAGGGAAACTAACTACTACGCTGTAATTTTATCAGGCGGGGACGACGCTTTGGGGGATAGAGACGTTGACAGAGCAATTAAAACGTTTTTAGAAAGCCGATTCCCTGTAAGTCTTAGCGATAATGACGTATCACAACTAAAGGAACAGGTCAGCAGGAATGGTAATAATCAATCTGTTACTGTATCAGGTACCAATGTCTGTCTAACCTATTCGGATCTGATCAGTATTATTAGACCTTTTTTAGATAGAGCTGGAAGAGTGTTGGCGGATGTCTATAGAGACAGTGGTTTACAAGGCGACATTACATTAGTACCCATAGGGGGGTCTGCTTTATTACCTGGTATAATAGCTTCGGCAAAAATGTATCTTAATAAAATAAGAACCGAGTTGGTATACCCTAGGTTGAGGACGGCAGTTAGTGAGGGTTGCTCCCTTGTGTCTGCCACAGTAGGGACACCTGGCTATCTATTTGTCGATTGCATAACAAGCACTATTAGCGGTGTTACGGGATTCTTTTGCGTTACACCGTTGATACCCAGAGGTTCTCCTCTTCCTTGCACAGCGACTAGATTGTACAAGACTTCTAGTAATTACAACGTCCGCTATCTCATAGCGTTTTTTGAAGGTGACAGTATTAGGGAGTTCAACAACAAGTTGATTACCAGATTTCGCATAGATAGAAAAGTGTTGGGCATAAATGTTAATGCTCCATGGTCATTCTCATCAAAAATATCAGTATCACCCCTCGGGCTTCTCACTGTTGAGATAGTGAGTGGCATGAGTTCACTGGTGATTAATAAATCGGCGCACGTACCAGTTTTTGACGAATTATCTTGTAACCTGGAACAGGTGGTTGTCCCGAAAGGTCAGTTATCAAGCGTAGCTTTAGCTGATTATAATATATCTCAGAGTATTACCAAAATTCCTAAGATTAAGGCAATCGACAATATAACGGCTTATTTACGGTATTTACGTGAAACGCAAGGTTCCGAATTTGCCGAAGTGGAGCTTAAGCATTATTACGGATCAGATGAGCAGATTACTGGTAAGGTCGGACTGGAAGTACGGCGACCTATTCCGATATTTTTTAGGGAAGAAGGATATTCAGTCTATCCTCGCTGAAGTGCAGCGTTATCCCAGCAGTTCTTTCAAGAGTTCTCACAATATATACAGACAAGGTGACAGGATCGAACGTATAGTATTAACAAACCATCCACAATACCCGAGATCCATTAAATACGATACCACTATTATAGCAGAGTACTTAATGCTATGTGCGTATGTTGAGAAGCACCGTATCGACAAATATTTAGATACTGAGACATTCGACATGTTAAGCGATATCATGTATGATTTTGATCTGTCAGATTTGGACTTGACTATAGTGCAATTGCCGAGGAAGAGACTGGAGGCGAATTTACAATTCAAACTTTCTGACTTCGACACGCCTGAGTATTCTCGTTACTCTAGTGATCAAAAGCTAATCTTTAAAACTATATCTAACGCAATGTCCGTGAAATATAATCCGCTGGTGTTGGTAAGTGGTGAATTAATTTATGCGGACGTCAAGTCCGACAACAGTGAAATACTCATCAACAACTACGAATTGCTGTGTAGAATGTACAAGCATGGTGAGGCTATAGCGTCGGGGGCCCTTAACAGTAGTTTTAATGTTAACGGTTCTCACTGGTTAAACAGGTTTATGAATAATTTAATGATAGCGTCAAAAGGGGTAGGTGCTTTCTATATAGGTGATCGGGTCGTACCTACTTTATGTTACTTAGGTCTTATTAGTAAGGTGTGTGATTATTACGACCCTTTTGGGAAGATTTGTGACAATGATAGTCAATTAGCTATTGTTAGCGAACCTCCCGTGTTAACGTGTATGTCTCTGATATTCGCACCACTAAATACATTTGTAAATGATGTTAAAATTCTACTACCTTTTTACCTAACGGCCGACGGTACTTTGTCGAGAACTTTAAACTATTCCGATCTTTCGTTTTTACCTGTTCAAAGATCAAGGTTAAACTTATTGAGCAAAGAATTCGTGAGAAGGATTACAGCTAACATACTGTATTTACCAGACTATGATGATTCCTTATCCGAGTTAGGAACCTATTGGCTGTTTTCGGCCGTCTGTATTTACTACGGAATAGAAGGTACCAACAGGTTCAGAAAGACTGCTAGGGGAAAAGGGGTATGTTTTTCTGATCATTCAGGGAAAACTTATTTAGTAGACATGACAAGGCTTGAAATGTATTTCGATGAATTACAAAAGGATATTGCCAGCTATAGTGTTAGAAGAGCTTACTTCGGTACAATAATAGAATTTGTTAATAAAATATACGACACTTTTAGGTGTCAATTTCTTTGCAGGTGGTATTATAATGGTTATGGCCCTATGTCATCTAAGGACTACACAGATTTTTTCAAATATAATGGTAGTGGTGTTGATGTCAAGTATCTTAAGTCAATGCGAGCGTACTCGGGCGTTGTGTCTTTACGGCCAAATCTCAGGGGAGCGACACGTCAGAAAGTTCGACGACGGTAGTTGTCTGGAACTCAACAGGTTGAAAGCAATGCGACTGTCATTAAGCAAAAATGGATCGGATGTTACTAAATTAGGCAATACCTTGGTAGAATGGACTTATGATAATTCTGAGTACTATTACGACATTTCGCTTGTCGACGGTTACTCTGCACCTATATCAGTGTATTGTGGCGGTGCGGCGATCAAATGGCCTATCGATCCGGTAGATTATTGCCCTACAGGGTTGAGTGATAACATTTGCAAAAGTCCTTGTACTTCTAATCGCTCTGATGTGGACTGTTGTGTAGGTGACTATCAGTCTCACGAAAGATGTCCACCGAACGATTGGAACAACGAATTATCGAAGATTACGACAGATGTGTATAGACAAGCATTTGATGATTTGCAAGCTTTAAAAACTTGTAATACTACGTTAACAGTGTGCAACGACCAATATTCTAAAATTGTAACTCCTAAGGATGGTAGTTCGCACGGCAAGTTTAATTCAACTACATCAAGTAGAGAAATAGATGTACCTAAGACGATGTCTTTAGTTGTAGTATATATGTTTTACTATTTAATAATTGTGAATTAAAATGACTACCAAGGAAACGAACAAGGCTAACGTTACGTCAACTTCATCAGACACTAACCATGATTTTATGTTGGGTGAGCTTGGAATAGACGTCGCCACACTTACTAAGAATGTAGAAAAAATAAAGAAGAAAGGATTCTTTGAGCTCAATACAAATAGAATGTATAACAAGGACCATCAAGATACCATACATAAAGGACTCAGATCGTCTATTCACAGTAAATACCCAAACCTTTTGGCTAATGACGATACGGTATGGCCGACATTGTTTACCCAGATTTTGTGCAGAGTCGCTATTAGGCAGACCTCTGCTAAGACTAATTACTCCGAGTCAGTAAACTATTGCGGCGGCACGGATTTTGAAACCGCAGTGGCTATTCCGGACAGAGATATTAGGAACTTCATTATCCAGGCAGCCCACGACGCGGAAACCCATCCAAACCCTGAGAGAAAGTTTTTCAGAGCTTACAGCGGTATGTGGCTCAAAATCTGTCAAGCCGGGGGGGATAAGGAACTAGAAAATACTTCCTTGGCGGCTAAGTGGGGATTACCTCAGGACTACAGGGCACTAACCCCTGATTTTATGGAAGCGACTAGGGAAATGAGTGATGAGTACGCTGAAGCTTTGCGTTTGAAAACTAGGGAAGCGGTATCTTCGGCTCCTAGTGTTGCTAATGCTCCTTTACTGAACACAAGTTTACTGTCTAGACAATTTACTTCAGGTTATCATTGATTAATCTGTGTCTATATGTAAAAGCTAATAAGTAAACACTATACCAAACTTGTAATTATGAATTTTAAAGGACACACACCGCCAAAGAAATTGGACGAAGCGTCCCAGATAAGCATAACAGTAATTACGAAGAAATGCAAATATTACGTTTCAGCGGAGGTCCACTGGCATGCTGATTTCTGGTTAATTTACTATGATGGTGAACACAGTTATTCTTACTTTTCTGATAGAAACACTAACAAGATAAGTAAAATTAAATTATTTGGAGACTGGTTTAATGTTATCAAACACAACAGTGTTCACATCAATTATGTGTCTATAATAAAATATAATCATCTTGAGCGACCTGACGATGAAAACGACCCTTGTGACCGTATTATCATCGATGATAAGATG

**> *Actinidia deliciosa virus* 1 variant 2 (AdV-1 v2) genomic RNA, complete genome**

AACCCACTCAGTTCCTCAGGCAACCCTTTCCGTCTTTCTTTTCCTTCTTCTTTTCATTTTGTAGCTCCTTACCACATAAAGTTCCATAAAATAAAATAAAAACACATCCACACAATCGAACATGCGGATCGTTCGTGCACCAAAAGGGTGCACTCGAAGGGTGGCACAATCTGGTGCCACCAAGTTGGGCCTCAAGGGTGAGTGCTCAAAGAATCTTCCCCGAGGGTGGGGGGCGATGGGGTACCTTAGTACCCCGGTATTTTTCGGGGAGGAGGACCCGGAGAGCACCTTTGTGCGAGGGTGCTCTCGCATCCTCGACTGCTTCGGGCTCAACTGCCCGAGGGCCGTCTTCTACGGTCCCGCACCAGCCACTCCAGCCTATCTGAAGGCTGCCGCCATCAGGGCGAAGATGGCCGAGACCGCTCGGCCATTCGGTTCTAGGGTGGCGTCGCGCCAGGCTGCAATCGCAGCTGATTTGTACAAGGCGCGCCAGCTTAGGGGCGCCAAGGTTCGCCCCGAACCTGTCCTCAAGAGGAGGAAGGTCGGACCGGCTTTTAAGCCGGCAGCGGTGGAGGCATCTGGCCCCACCTTCGTTAGAGTTACGGCGCCGGCTGTTCCGGCGGTAGTGCCGACTTCCTACCCCGTCGCCTACGTCAGGCGCGGGGCTCAGAGCCTACCTCAAGGGGCTCTAGTCTTTCAGGCTAGGAAGCCTGCTAACAGGACCATAGGGTTCTGTGCCGCGAAGCAGCGGCAGACTACCACGCGGCTGAGCGTGGGAGGTTGGACCGTCACCCTCCGCACTGAAGAGGGTGCCGTGACGGATGAGGCCCTCCTGCACCTGGTTAGGGGGTACCACTTTGAAGCCTTCCTACCGATGAGGCTTTTAGCCAGGTACGGGGCTAAGTTGACACCCACCTTGGACTATGGGTGGGTGCGGTACGACCGTAGCGGCTACCAGAGCCGTTTGATGATGCTCCCTTACCTTTGGGAGGTCCTCCAACTTAATAAGAGGGGGGAGTGTGGGCCCAAGCTCAGGGCCTATATTGAGTCGTTTAAGGATAACAGTGGCTACTGTTACCTTAAACTTTTCAGAATGGCTAATATAGCCATAGGCAGATCGGCCAGGAAGGTCGGTCACGCTTGCAGTGCGTTGGGGTCTTTTCCTTCAACGCACGCTGTTAAGATGTGCCTATATAGGCGCTACTCATGTATACCTGACTTTTCTGTCGGGTACAAGGCTGTTGGTGGCAGGGGTCACATGACCACAACCCCTGTCGTGAAGGTGTCATCTCTCCCGGATTTGGCGTGGGGGTGTGACACTCTCATGTTTGGTTCCATACGGGCGGTTATTCCGCCCGTACAAAGAGCAGTGGTGGCCAAACCTACCGCAACTGCGGACAAGATAAGTCAGGTTCCGCCACCCAAACCTGCGGTAGCGCAATGTAGCGCCAACGTTATTGCTGTCGCGTCGACGGGTGACAATTTAGTCATGTCGAAATCGGCAAGGCGCAGGCAGCGTAGGCGCTGTGCGGCTGTCAAGGCCGTTCCGGCAGTCCCGACAGGCGAGCACAGGACGTTAAAAGCGGTGGAAGTTGACGCCGCGCGACCCAGGCCAGTCGCTGTGTCGACGCCTGTTAAGGTGACTACGCCCGCCCCGCGGATCCACCAATTCATTAAGCCGGTCTTCAGAGATATTAAACAGTCTGGGAGTCGGCTTAGGAACTGGTTTGGTAAGGTCAACCCGAACTATCGGGTGAAGGATAGGCTCACTGCCCTCCTATCGGCAGACGGTACGGGTTATGAATACAGCGGCGGTAGCCATAAACCTGATCGCCGTAGCGCGACCTTGCTAAAGGAACTGTCTTCTATACTGAAGATGGATTTAGGTTGGGTCAAGCACGTCTTGGTCCAGAAATACAGACCCGGTTCAAAAATAGGTGCTCACAAGGATAATGAGCCCTGTTACTACCCGCTGAGCACGTTTAGGCTTGTAACTGTGAACGTGTTTGGGGAAGCTTTGTTTACCCTCGTCAGAGGCGCTGAAAAGTACAATATAAGCTTGGACGGCCCCTCTATGTTCGAGATAGACCCAAATGTCAATTTCAATTTCACACATAGTGTAGAAGTTGGGAGATTTTACAGGGGCTCTATCACCCTTAGGGGACATAAGCAGTCGAGTGTCCTGGGAGAAGCGACTCCAAAGCTAACTAATGACGCGTCATCGGTTACGACCAAGACAGTCAGGACTGAAACTTCCCAGGGCGGAGCCGGGCTAACTGCTCCGTCTATCAAAGGGATTATTCCAACTGTGCCCCAGGTTAAGGTCAGCGCGACTAGTCCAAGAACTAGTTCGGCCGTGGTGGCACCTAAGGCTATTGCGCGAAGTCCGACCATCATCGGTCGCTCTGCAGTAGCCACTAAGCAGGCCACCACAGGACAAGGCACTGCAGTTAGTGTGACGCCAACTGTGGACCATGGAGTGTTTTTAAAAGCTCTAACAGAGTGTTTTTCAGTTGGCGAACCTTATGATAGGGTAAAATTCAAAGATTACAACCCTGTTTGCCAAAAATCTGAATATGGGATGGTCCACATCTTTTATAAAGGGAACTTAGTCAGGAAAGGCACTTTTAGGAGGTACTATGATTTAAAAGCTCTCAGGCAATTAGGTTATGTCACTAATAACCTTAGGGCGTATCTGAACTCCTTTAGGGACAGTAATGGTTACTGTTATTTGACTTACATCAGAGCTGTAGCAATGTATTTCGGTTTGGCGGAATCTGAGTGCGCCGCTGCTACCAGGGCTTTGGGACGATGGCCCAGAGCCGGTGATGTGTTAGCCTATATAACCAGGAAGTACAAGGTTTGCCCGCCAATTAAGGTAGGGTACTCACACGTCACTAATTTGGCAGTACATGCCACTTTGGAGCCAGTTTTCCTCCTAGCCAATATGCGTAAAAACCTGGTTGTTGGGGGACGACTGGCTGCTTCTGTGTCTTCCCGCGGGATAACCGTAGGTGCTATACAGTGCCCTATTCGGAATCCAGCAGGAATAAACGCGGCGTTCGAGTGTCAAGTGGAGACATCGCCGGCGGATTGTAGTGCAACTAGGCGGACCAGGTTAACTAACGTTGGTTTGGGTGCTAACACAAGGGTGGCACGTCCTGTCTGCCCTACCAAGCAGAAAGTTTTTACCAATGAGCTTAAAAGCAGTGAATCGCTGGCCAGTAACAGTCCGAGAGGAGAACCTGTACTCATTGCAAGTTCTCGCAAGGAACATAGTCAGCTGGCCAGTGAATTGGTTGACAGAATTTTGGATTTTGACCAAACGCAACCGTCCCTGAACATCGAACGTTTTAAAAACTTTGATGTTAGATGTGTGAAGAGTAGGCCCGGTCTGGTCAGGGTTTACTATAGAGATCGTCTGATAAGGACGTTGACAGCCCAGCGCTATTGGGATATAAATTTCCTTCGATCTTTTGGGATTGTAGCGAGAACCTTAAATAATTACTTGCGAAGGTTCGGTGATTGTGACGGTTACTGTTACATGAAGCTATTGCGGTTATGTAGTATTTACTACGCCAAACCAATGAGCTATGTAAAGTCCGCAAATTTAGAGTTAGGGTGTTGGCCCAGCTCCAGTAGTGTTAAGGCGTACATACGGAGGAAGTTTAACGGTATACCTTTTATACTTGTATCTCTTTCTAGAGGGAAGTATGCTCATGTGGGGCTCCTACCTAGGGTATCTTTGAGATTGATACCCGGAGGTATTAAATTAGGAGGCCACGTGGAGGCTGACGCCTCTTTAAGGCGCATGACTGAAGTCAATAGATTAAACCAACAAGTCGAAAAGGCCCAGTTGAAAGACTCTGCACTTCCTAGGGCAGTGGAGAATACTCTGATTGAAGAGCATACTATGGAACATCTACTCCAGAAGTCAAAAACTAACCTGAACGTTAATGTGGGTTTGAATGATCGCCAGCAGTCTGCTTTGGTTAAGAGTTTCCCCGAATTGAAGTTAAAATTTGTACCTATGGTGCATTCATTGCACCCGATGAGCACCGCTGTGAGAATGTGCTTCAATAGTTTGTACGCTAGGAAATTCAATGGAACCCAGTATATTGATATTGGTGGTGATTTGAAGTACCACGTTACGCATGGTAACAATGTCCATATTTGCAATCCTATCCTTGACCCTAAAGATGGAGTGAGGTACGTTAACAGGGTTTGTGAATGGAACAATATTAAGAAGGCCGACTTGAAAGCTATGGCTTACGGTTCGCAGCATGTGTCCTGCTGTTACAGTCCCGCGAAGATTTGCAAGGTGTCATGCAGCACTGCTGTTGCTGTTGAAGTTTATGACATAAGCTTAGTCGAAATGGCTACTATTATGTCGGCCAGAGCTATAGACAGAGTATACTTAACCATGATGGTACCGGGTGAGTTATTCGATGATAATAACGAACATGTATGCATCCCAGATCATGGTATAGTTGTGGTTCAGGATGGGGATAATTTAATATATAACATGCCCGCCGGACAGAGTTATTGTCATGATAGGTCAAGTGTCTTATCATACATTAATAACCCTTATATGCTTCATGATAATCAGTTGTTTCATTCTGAAATTGTAGGTTATAGGTGTGGTGTGTGTGAATTCAGGGTTACTAGAGTACCTGTCTACCCTGCTGTAGATACCGTTATTCACGTAACTGTTCCAAGAGCTACTTCGGGGTTGGTGGAGTTGCATCTTCCCATTATAGACAAACACTCTGATGTTTTAAGCTTTACTAACTCTACATCAGTTATGATAGACTACGAGTTTTTTACTAGGGCGTTAACTCACGTTATTAATGTGTGTACTAACGTGACTGAAAAGACCTTCGAGTACACCATGACTTGGCTGAGGAACAATTCGGCCAGAGTGGTCATATCTGGAAGGATCATTCATACTAACGTCAAATTAGCCCCAGAGCATTTGGGCAAGGTGGCAGCACTATTACTTACGGCAGGTGTTAAGACAAGGTGGGAGAGCGGTAGATATGCCAGACGATTATATAGGGCTGTAGGTCAGGAGACGCTGTGGGAGTCCATAAAGGCTGCCATAGACGACTCCACTTCGACTATTAAAGCATCGGCGTATGAGTTAGCAAAAAAGGCACTGACGAGTTCATTCCCTTTCTTAGGGGACTTGCAGACCAGGTCTGTAGAAGAGTTCTTTACCGTCCTAGGCGAGTCTTCGACTATAAAGCTTCCAGTGCGATTTCCTTGCAGTGGTGGCTATATAGTCGGGGAAACGAGATACATCTCCGAAGCTATGGACATCTTCTAGCCGACGCAATCAGAAGCGACGTGCTGTCTGAAATGGCTGAAATCGTAGAGGAATCTACGTCTGTCGACGCGTCAGGGAAGGGTCAAGACAACTACGTTCCCCCAAACAAGAGGGCTGTGAAAAGTGCTGGGATTATCCGCGCCAAGGCAAAAGCTGTGGCGGGTGTGACTGAACTGTGTGACGGCTCAAAGCCTGGTCAAGGCGCCGGCAGGGATAGTTGCGGATCGTCTTTGTTTGTAGTCATACTAAAAGCTATAGGACGCTATATTGTGTTAACGGTATCTCGGTTCAAGGAGATATTATTAAGGTTAACGTCTCCTTTCCCTAATGTGCAAAATGTGCTTTGTCCTGTTTTTGATCTGTGGGAAGGTTTATTAGGCGGGGACGCAGATGTATGGGTTACTTACGGAGCCACTGTAGTGTATACTATGGTGCGCTCTGTGGTATACTTGGCGCTAGGTCACTCGCCGTTTGGTGTCTGTCTTGGTCTATTGGCGGTAATAGTAACTCCTATTCCCCGTTTGTTTGTGACAGATCGAGACAACTTAGCCTGTGACACTCTTCTTGAAGCGGTAAAAGGAGCTTATTTTTCAGTACCTTTAACAGGCAACAAATGGTTGAATAGAGTGTTGTCTGTATTGGAAAGTGTCGGTTATTTTAAGTCTCTGATTAGGAGGACGATTGCTGTAATGTTTGAAGAGAGCACTGCTGCCTCCGTGGTCATATTGGTTATGCCCGCTGACGAAAATATATGGGCGACACAGTGTTTAGTGGGGAAGGCCTATGACTGGATTCACTGCCAAGTTCTAGCTACCCTTAACTCTATGTTGGAAGCTGTACCTCGCTCAGCGAGGAAGCTTGTTTCTGACGCGGTGGAGGATGTGGCTGGTGGTATATCGTCTGTTTTGGCCACGTCCGTAGCGAAGGTGACCGAATGGTTCAGCCACCAGGGCATCACCTCTCTGCCAGCTTGTGATGATGAATCTGTGGCTGATTTCTTCAGCATGGCTGAGGATACCAGCTTGGTGGACGACTTGACGTCGGACACGCCCGGACTTAAAGGGGGGGCGGTTCTAAATAGAAGTTTCTTCACTTCGTTGGTCAAAGCTTTCATTAACACGGGTCTAAGTCTTTTTGAGGGTATTATCCAAGCTGCTATGTATGTGAAGGATAAGTTGTATCCCGGGTCAGGTTGCAGGACCAAAAAAGGTAGCGAATTGCTACAAGAATTATTCTCAAATCAAAAGTCGGAAGATGAGGAATTCGAATTAGCGGAAATTTACCTACGAGAGTTCTTTGATGTGGACCTGTCGGACCGTCCTGGTGCTTATGGCGGGTCCGTTGAGTGTAAGTCCTTCGCTTTAGCTGTTTATAGGTTTTTAAAATCGTTTAAATTTTGCAGGGTTATAGCGTTGTGCCAAGCCATATTGGTTTCTCTTGTTATGGGGAAAAATATGTGTATGAAACAATACAGAGATGCAATGGCAGTTGCCCGTCATTTGTTAGCTGTGTACAGGAAAAAGAACCCCGGACTAGCGTTGGCTAAGCTAGCCGATGTCGACGTGGATGATAGTAAAGTTTATACAGTGCCGACGGATTTATTTGATGCCGACGAAAAAATAAGATCGTTGTCTAAAAATTGTAAACCTAACTACTTCTTAAGGAAAGACATGACTGTTTACAGGGTGCCAGCTGCGATTGACTACGAGGATAAAAATGGGTTGGCTGCTAACCTTGTTTCAGCTCCATTTGATTTCTTGATGTTCCGGGATGATGCCCCACCTGAAGCTGTAAGCTTGGATTTTAATCTTATTAAGAGGATGTTAGGTTGGACTATGACTAAAGATTTAGTTAGTAGGATTCCTATTGCTAGGAGACTCCACCCGAGCGCTATCTTGATGGTGAGAAAAGGTGGTTATGCTATATTTGATAAGAATAGGAAACCAATTGTGTATAATTGTCCAGACGTAGGTTTAACACCTGAGAAAATGGACGTGATTTTTATGAGATTGTCTGGCGGATTGCTGGGTGGGGGTGTTGTTTCGTGCAGTTTATTACTAATCCTGAGGGGATTTTTTAACGCCCTTGAAAGATGTGGTATTATAAGCAGACACTTGAATACTGCTTCAAAGGCTATTTGTTGCACTTTTTCCCCTTTTTACAGGCTCGTGGTTTTGCTGCGTTGGTTGCTCGACATTTCATGTGATTGGTATCAAGAGAATGGCGGCAATCGCAATGAAATGGAAATCAAATGTTTAAAACCTGTAACCAAAAAATTTGTTGCCGTTCCCGAGCAGTTAAAACGTAAATATAATGATGTTCTAAATAGAAAAGTCGAGGACGTCGACGTGTTGGCGGAAGAGTTAGTTCGTCAACCAACTGACGGGTCGACTGACGGATCCAGCTCAGAGATATCGACGTGCAATAGCGACGGCGAAGAGTATAACCCTAGATTCTTCAATAAAAAAGGTAAGAAATCTAAGAGTAACTCTCGCGGGAAACGTGTTTCTACAGTCAGAACGGCAGAGCAGCGCAAACACTCAGCTACAGAGCGTGGCGAGGCAAGCGGTACCCAAGCCGAAGTGGTTGAACAGCCAGACTCTGATAAGGTGTTCAAGCCTATAAATGTAAATGATGAGTTTAAACTAAGGATGGGCGATGTGTACAACAGGAAAGAGTACCAAATAGCTGAAGTTATTCAAGCCTTGGACTTGTGCACTCCGCCGGTTTTTAGTCACACTTCGGATTTGGCCACTAATGCTATGAACGAATTTGTCTTTATGCATATGATGAATGTATTAAACATGCTAAGTAGTATGCAAAAGGCGTCTGAGCTTTTAATGGGTGGTACTAGAGATCCTGCTATGTTGCGGGGTTATATGGTAGATCCTAAGGTAGTCATTCTAGATACGTCGACGAACATGTTGAGAGGTACCAACATTGGGGTAAAGCATATAAAAGACACTCAGTACAGATTCTGTTATGATCCTAACAGTAAAACTGTAGTGTCCCTAGGTAGTTATAGACTTAAGTCTTGCAACCAATACGTGATACTCCACGAAGATTTAGAGGTTTTTTACGCTAATAATGTACTCCAGAGGTTCGATGCGAGCATGAAAGTGCAAAAGATGCACTTCCTCAGTGATTTGACTATAGTGGAAACTCCTCCAGGGGGGGGAAAGACTACACAGTTAGTAGCACTATTCTTCGGTCTGTGGATGAAAGGTGCTGCAGTCAGAGTTATCACTGCTAATAGGAACTCGGCTGAAGAAATAAGACGTAAAACATGCGCTCTAGCTCTTCACTTTAAAGTTGTGACCAATTTGCAGCTTGTTAGGGTGCGTCAGCTTTTAGAAGATATGGTGAGAACAGCTGATTCCACAATAATGAACGTGCGAGATGCTCCGACTGAAGTTCTATTAGTTGACGAGATTTTCCTGATGCATCTCGGTCAGTTGTTGTTGAACTTTGAAATACTCAAACCTAACTACGTTATAGGTTATGGGGATTCTAAACAGATAGCTTATATACCTAGAACAGATCTTTACTGTCCTTGTTATTATAAAGTTACGGATATAATTGATGATGATCAAATACAGTTCAGGAGTGAATCTTATAGGTGTCCTAAAGACGTGTGCCTGCTACTTTCGGAGTTGTACGGTCGTCACGTAGAAGCTAGGGTTAACAAGAGGGACAAGACTATGACAGTGACCACCATAACTTCTTTGGAGGATGTTCCTTTAATAGAGGATGCTAAGTATTTGGTGTATACCCAAGGGGAGAAACGGGATTTGGATGCCGTTCTAAGAAGGAAAGGTAGATCGCCTTCCACCTATCTTGATCCTCAGACAGTCCATGAGGCGCAAGGTAACACTTATAAGAAAGTGTGTTTAGTTAGGGCTAAACCTCAGGATGATAGTGTGTTCTCTTCAAAAGAGCACCACATAGTGGCGTTGTCTAGACATACAGATTCACTGGTCTATTATTGCATATCATCAAAGTATAACAATGATACAGCGATGAAGATAGAGAGATCCAAAGTTCTAACGGCGCTCAATGATAATGAAATAAATGAGCAACCGATCTACGGAGCCATGTATGAAAGTAATGGCGGTAATCCAGCTTCCGGGGCTTGTAAGGCTGGAAGCATGGGTTGGCATGCGATTGTGAGTTTCTTGGATGAAGTTGTGCCCGGCTCGACAGTTCTATCTTTGAACGACGTTTCCGAAGCTATGTCGACTTCAGATTTCGAAAGTTGTGTTGACGAGATTAGGTTGAGTGAGAATATGACAGTCGGCAAGAATCCGACTAGCACGAACTGTCAGCGTTATCGGCGTCATAAGGCCGCAGGCCGTTCAGGATAGAAAACCTACATTACAGGAGAACGTGTACAGTTATGAAGCAAGGAACTTTGTGGCTTTAACTTTAGATAGGCACCTCGATCCTGATCTGTTCAGGGATTACGCTGTAGATAAATTTTTTAGAAAATGCATCAACATGGAAGTGTTACAAGGACTTAATTCTTCTGTAGTGGTCACAAACCACCTCCATTTCCAAGAGTGGTTAAGGAAGAGGGATGGTTCTGCCTTAGCTAAACTTGATAAGGAAGTTAATTATATCACGACATGGAGGGATTATATGTCACTCTTCAAACTCATGGTGAAGAAGGAAGCCAAAGTCAAACTTGACGCTTCATCATTGACTAAGCACAACCCTGCTCAGAATATCATTTTCCATATTAAATTTATAAATGCCGTTTTCAGCTCTGTATTCGCCCAATTGTCCGAACGGTTGAGGATGGTGTTGAGGCGGAATATCATTCTTTATACTAGTATGTCTATAGATAAGTTTGCAGACTGTTTATATGACGTTTTGGGCGGTCGTAATGTATACAACACTGTGGAAATGGATTTCTCAAAATTCGACAAATCTCAAGACGTATACATAAAAGCTTGCGAGATGGAGATATACAGGAGACTGGGTATGTCCGAAGAGTTGATAGACCTGTGGACCGCTGCAGAAACCTTTTGTAAAGCTCAATCTCTGGACAAGGATGTGGCGTTTGATGTGGGCGCGCAGCGGAGATCAGGCACAGCTAACACGTTTTTGGGGAACAGTATTGTTACCCTTCTATTGTTGTCACAGTATTACGAAATTACAGATTTGAATTGCTTAGCTGTGGCCGGTGACGATTCCATTATGTTTGCGGAAGCGGACCTAGTTCGTGCTGATCCGGAAATTCGTACCCGAGACGGTAAGGGTACTTACGACCTTCTTTTCCATAGGAAGGAGGTTATTCCTGACTTTAGTCATGAGTTAATGGTGGATCTTGGAATGGAAGCCAAGCTTTTCAGAGACCTACCAGCCTACTTCTGCTCGAAATTTATTGTTTTTTGTAATGAACGCGTGTATGTGATACCCGATCCGTATAAACTTATGGTGAAGCTAGGTAAACCGTACAATGATTGGGATGACTCTGTTTTGGCTGAAAGGTTCATATCTTTCAGGGATCACACCAAATATCTCGATGACGAATGTGTGGTATCAGCCCTTACCGAGGCAGTGAATATAAGGTACAATCTGGTAGGTTATCACACTTACGCAGCTATTAGCGCCTTGCACTGCGTTTCAGCTAACAAAAAGAGGTTCTTTGAATTATACCCTTTTAAACAAGGGTTTATTACTCGCTATGTCAGGAGAGTCGGTAGGGTTATTAGGAAATTCAAGCAATCACTCAAGGCTGCAGGGCTTTTCATCTTGGATAAGAGAAGTCACGAGCATGAAGATGTATATGATTGCGCTTTCAGGGACGTCTATGAAGCCAGGTTAAACCCTGATGTGTTCGGGCAAGTAATTTAAGTTCTCCGAAGGATAAAGCGGAGTCCCACCTGTGCATTTAGTCGCTAAGTTCACCATATATTTTGAACGGTCAAATCCTCCTGTCAGAGGAGGGCCGGGAATACTCCGGTCTTTACCGACCTTCAATACCACTCTGTAGATTGTATAATTTATATGTGATGTCTGTGATGAAAGGATGGGGATAATAAACTCGTTGTACGAACCTAGTGACACTTTGCGAGAGTATAACCAATACAGTCAGTATACTGGAAGTGGTCGATGGTTACCACACACTGGATACGGCGGTTCTCAATATGCCTCTAATCACTTAAATGGTGGTTTGCGCATGGTTGGTAATCCTTCTATTGATGCATTGATGATACAACATAACTATAAGGAATTAGAGATCTTGTTAAGCGAAAAGCGCAAGATAACCTCTGAACACGCTATGGAGAGAGAAAACTGCTATAAAGAAGAGATGCGTAAGCGCAACGAATGTTATGCAAGGCATAAGAGAAGGTCTAAGAAAAGGTTTAGCTTATACTCGTTGTTGTATCGTTGACAGTATTAATCTATATATTTAGTAATGGGTTCTCCGTGGAAGTTCTTTGATAACTTGGACTTTTTGGTGACGGTTACAAATTCGGTGCTTTTGGGTCTTAAAACATATTGTTATTTAAAAAGCTCTGTTACTGTACTTTTAGTGTTAGTGATAGATAGTTTAATGGATATGGTTTTCCAGTTATTGAATTGGTTGACCAATGAGTGCGGTAACGATTATATCTTGGCCGTCGCTTATACGTCTGTGATGCTAACGCAATTGTATCCGTTGCATTTGGTGTCTATGTCTTTACTCAATTTAGAAGACTACGGTCAAAAGAATACAAGAGTGGTAACTATAATGGTTATACTAGTACTATTGATACAAATCTTTTTAAAATCTGTGCTATTCGTAGTCGGCAAGATAAGATATAATCTAGTAAGAAATTTCTCTCTGATCGCTGATCAGCTATATGATATGTTAACTACGATAATTTCACTTGTTTTCTTCATACTGATTGCACATTTTCATGCACCTGTGTCTAATTTATTAGATTATTGGGGTACCATAGTTTTAGTCTTGTTGACGTTCTTCTTTTGGTTAAACAATTGGTACGAGAGAGAAAGGGTCGTTGGTAGACCTGATCCACAAGAGAGTCCTAGTTCGGAATTCGTATTTCCTAACCATACGTCCAATGAACTAACTGTTGAAGTTTGTTAATGGATGAGTTGTTACAGGTGGCATTTTCGTTACTCTTCCTTTTAGGGTTTTGTCTACTTATGTGTACTTGTTTAGTTGGAAGTTTCTCAGTTTACAGAAAGGTGAGTGACCCGGATACCGTGGCAGAAAACGCCAATAGGTTGGGTAGACTATGACGATTATAGGTATTGACTACGGTACCACGTTTTCAACGTGCAGTATAGTCACATCAACTTCAGTTTTTATACTCCAACATAATGATTCAGAATACATTCCCAGCCTTATTGCAATAAATAATGGTACAGGGTCTGTCGTCGTAGGAGTTGATGTTATCGAAAAAGAGCAGAATACATCATATAGTTGTTACAAGGACATGAAAAGATGGGTGGGAGTAGATGATTCTTCTTATAGCGAGAGAGTGCTCAAGCTTAATCCTACGTACGACACTAAATCTCACGTAGATATGTATGATTTCGAACTTGGCGCTTACAACACTAAAGGTAGGCTAATGCCCATCAGATCATTAATTTCACTCTACATAAAAACTTTGGTTAAGTTATTTGAAACTCGTTGCTCTGTTGTCTGCAGCGGTTTAGTTCTTTCAGTACCTTCTCAGTATACTACATCGCAAAGATCTTTCATGGTAGCGTTAGCCAGCGTTATCGGAATAAAAATAGTTCATATTATGAATGAACCGTCTGCCGCATTATTCGCATCTGTGAGTAGTATACCTGATAAGGTAGCTAGTGAGTACTATATAGTTTACGATTTCGGTGGAGGGACGTTTGACGTTTCTATAGTAGGTAGGGAAACTAACTACTACGCTGTAATTTTGTCAGGCGGGGACGACGCCCTAGGGGGCAGAGACGTTGACAGAGCTATAAAAGATTTTTTAGAGAACCGGTTTTCCGTAAAAATTAAGGACAATGATGTGTCGCAACTTAAAGAACAAGTTAGCAGGAACAGTAACAACCAATCCATAACAGTGTCGGGTGTTAACGTCCTCTTAACTTACTCAGATTTGATAAACATTATCAGACCTTTTCTGGATAGGGCCGGAAGAGTGTTAGCCGATGTCTACAGGGACAGCGGGTTACAGGGTGATATTACTTTGGTACCCATAGGAGGGTCTGCTTTGTTACCGGGCATAATAGCGTCAGCTAAAATGTATCTTAGCAAAATAAGATCCGAGTTGGTATATCCAAGATTAAGAACAGCAGTAAGTGAGGGTTGCTCCCTCGTGTCTGCTACTGTAGGGACGCCCGGTTATTTATTTGTTGATTGTATAACGAGTACCATTAGTGGCGTTACAGGGTTCTTTTGCGTCACACCGTTAATACCCAGAGGTTCTCCGCTTCCGTGTACAGCTACGCGATCGTATAGGACTTCAAGTAATTATAACGTCCGTTATCTCATAGCTTTTTATGAAGGTGATAACATCAGGGAATTTAACAATAAGTTAATCACCAGGTTTCGTATAGATAGAAAAGTGCTAGGTATAAACGTTGATGCCCCGTGGTCATTCTCGACAAAAATATCGGTGTCGCCGCTCGGGCTACTTACTGTCGAAGTAGTGAGTGGTATGAGTTCGCTAGTTATTAACAAGTCAGCGACTGTACCTCTCTTCAGTGAATTGCCATGCAACCTAGAGCAGGTTGTTATATCCAAAGATCAATTATCAAGCGTTGCTTTGGCTGATTATAATATATCTCAGAGTATTACTAAAGTTCCTAAAACCAGGGCAATAGATAATATGACAGCTTATTTGCGATACTTACGTGAAACGCAAGGTTCTGAATTTGCTGAAGTGGAGTTCAGGCACTACTACGCTTCAAATGAGCACGTTACTCGTAAGGTCGGGATGGAAGTACGGCGACCTATTCCGATATTTTTTAGGGAAGAAGGATATTCAGTCTACCCTCGCTGAGGTGCAGCGTTACCCTAGTAGCTCATTTAAAAGTACCCATACCTTGTACAGACAAGGTGACAAGATCCAGCGTATAAGGCTGACCGATCATCCGGAATATCCGAAGTCGATCAGATATGATACCACTATAATAGCTGAGTACTTAATGTTATGTGCTTATGTTGAGGAACACGGCATAGATAAATACTTGGACACTGAAGTATTCGATTTGTTAAGTGATATCATGTATGAATTTGACTTGTCGGACTTAGACACGACTATAGTGCAAATGCCGAGGAAGAAATTGGAAGCGAATTTGCAATTCAAACTTTCCGATTTTGATACGCGCGAGTACTCTCGTTACTCTAACGATCAAAAATTAATCTTTAAAACAATATCCAACGCGATGTCATTGAAGTATAACCCACTAGTGTTAGTTAGCGGTGAGCTAGTTTACGCGGATGTCAAGTCTGACAATAGTGAGATACTTATTGATAACTACGAACTATTGTGTAGGATGTACAAACATGGCGAGGCTGTCGCGTCAGGTGCTCTTAACAGTAGATTCAATGTCAACGGTTCTCACTGGTTAAACGGGTTTATGAATAATCTAATGATAGCGTCAAAAGGAGTATGAGCTTTCTATATAGGTGAGTGGGTTGCACCTACTTTGTGTTATCTTGGTCTTGTGAGTAAAATGTGTGACTATTATGATCCTTTCGGAAAGATTTGCGACAATGGCAGTCAATTGGCCATTGTTAGCGAGTTACCCGTGCAGACGTGTCTGTCTCTGATATTTGCACCGTTAAATACATTCATAAATGACATTAGATTTTTGCTGCCCTTCTACTTGACAACCGATGGTACTTTATCGAGAACCCTAAATTATTCGGATCTCTCATTTTTACCAGTTCAAAGATCGAGATTAAATTTGTTGAGCAGAGAATTCGTAAGAAGGATCACTGCCAACATACTATATCTACCCGATTATGACGATTCGTTAAGCGAGTTAGGCAGTTTTTGGTTATTTTCAGCCGTTTGTACCTACTACGGAGTAGAAGGTACTAATAGGTTCAGAAAAACGGCTAGGGGGAAGGGAGTGTGTTTCGTAACTCACTCGGGAAAAACTCATTTAGTGGATATGACGAGGCTTGAGAAGTACTTCGAAGGATTACAGAAAGACATTAGCAGCTATAGTGTTAGAAGAGCTTATTTTGGTACAATAATAGAATTTGTTAACAAAATATACGACACTTTCAGGTGTCAGTTTCTTTGCAGATGGTATTACAATGGCTATGGTCCTATGTCGTCCAGGGATTATACGGATTTCTTTAAATATAATAGCAATGGTGTTGATGTCAAATATCTTAAGTCTATGCGGGCATACTCGGGCGTTGTGTCTTTACGGCCAAATTACAGGGGAGCAGTACGTCAGGAAGTTCGAAGATGGTAGTTGTCTAGAGCTTAACAAGTTCAAAGCAATGAGACTATCATTGAGCAAAAACGAATCGGACGTCACTGAATTGGGCAACACCTTAGTAGAATGGACTTATGATAACTCTCAATATTATTACGATATCTCGCTAGTGGACGGTTACTCTGCACCTATATCAGTCTATTGTGGTGACATGGCGATTAAATGGCCTATCGACCCGTTAGACTATTGTCCTATGAAGTTGACGGATAACATATGCAAGAGTCCTTGCACTTCTAATCGTTCTGACATAGACTGTTGTATAAATGACTACCAGTCTCACGAAAGATGCCACACCGAATGATGGGAACATCAAATTTATCGAAGTTAACCGTAGACGTATATAGACAAGCTTTTGATGATTTGCAAGCTTTAAAAACTTGCAGCGTGACTCCAATGGTGTGTAATGACTTATACCAGAAACCTAATATAACTCAAAAACCTGACGGTTCACGCGGCAGGTCTAATACGACCACATCAAACAATTTAAATAATTCACCTAAGACGATGTCTTCGGTTATAGTATACATACTTTGCTATTTGATAACTACATATTAAAATGACTACTAAAGAAACACAGAAGTCTAATGTCACGGCAGAATCTTCGGGCACCACCCACGACTTTATGTTGGGTGAGCTGGGGATAGATGTTGCCACACTCACTGCTAATGTTGAAAAGATTAAGAATAAGGGGTTCTTTGATCTTAACACCAATAAGATGTACAATAAGGAGCATCAAGACGCTATGCATAAGGCGCTCAGAGGATCCATTCACAGTAAGTATACAACTCTTATGGCTAATGACGACACTGTTTGGCCGACCTTGTTCACCCAGATTTTGTGCAGAGTCGCTATTAGGCAAACTTCCGGTAAGACCAACTACACAGAGTCTATAGATTACTACGGCGGTACGAACTTTGATATCGCAGTGGCCATTCCGGACAGAGATATCAGGAACTTCATCATCCAAGCTGCTCACGACGCAGAAACTCATCCAAACCCTGAAAGGAAATTCTTCAGGGCTTACAGCGGTATGTGGCTTAAAATCTGCCAAGCTGGAGGGGACAAGGCACTGGAAAATACTTCTCTGGCAGCTAAGTGGGGTTTGCCTCAAGGCTACAGGGCATTAACTCCTGATTTTATGGAGGCAACCAAGGAGATGAGTGACGAATACGCCGAAGCCTTGCGTTTAAAAACCAGGGAAGCTGTTTCGTCAGCTCCTAGTGTGGCTAATGCACCCTTGTTAAACACAAGTCTACTATCTAGACAGTTCACTTCGGGTTATCACTGATAATTCCGCATCTATATATTAATACTAATAAGTGAACAATATACCTAGCTTGTAATTATGAATTTTAAAGGACACACACCGCCAAAGAAATTGGACGAAGCATCCGAGATAAGTGTGACAGTGATCACGGAGAAATGTAAGTATTACGTCTCAGCGGAAGTTCACTGGCATGCTGATTTCTGGTTAATTTACTACGACGGCGAACACAGTTACTCTTACTTTTCCGATAAAAGTACTAATAGGATAAGTAAAATCAAATTACTTGGGGACTGGTTTAACGTTATTAAACACAACAGTGTTCATATCAATTACGTGTCTATAATAAAATACAGTCATCTTGAACGACCGGGTGACGAAAACGACTCTTGCGATCGTATCATCATCGACGATAAGATGCTCTGTGTATATAATAAAAGAGAACCTAAAGTTAATAAGTATCATTTAGTTACGAAAATTAATCCTTTAGAGGACATTCTGGACGACGTCGTTAGCGTCGATAGTTATTGTGTTATATACAAAATACCAATTTAAGTATTGTTATCGAAGATTATACTGATTCTTATTATTATATAGTCATATAAATGATTAACCGATTTAGTACTTAAATGGCGGAGTTATTATGGTCCTTTGTCTTGTTAATATTACTAATTGTTATGTTGTTAAGCGGTGCTACGTATGGGGTATTATATTACAAATCAGATAAAAATTTGAAATTATCTGATTCCTTGAGTTACGTTAAGGAAGGAGCTTTAATAATCAGTCCGTTACATGGCAATGGAATCGGAAATAGTGGTAACAGAACGCCAGTTATTACTTGAGAGACTGTCCACTAATGTGGTGGACAACGCTAAGGATATTACCAGGATTGTTGATGAACCCACTAATCCTGATCCGTCGAAGTCGCATTCTGTTTACTACAAAATATTACATTTTGCTGGACATGACGTCGTAAATAAAATCGTAAATACGCATCTTCGAGAATCTCAGCTAGACCTTTTGGTCGGTAAGAGGATCGAAATGATCAGTGAATACCAGATCACAAGGTGCTTGGTGACAGCGCTGGAACCGTCAAGGTACATGGCTTTTGGCAGTGCCACTATGTACGAGGATATGATGCGACTTAATAGCAGCGATTGGACTGCTACAGTTGTAAATGATGAAATCTTCGGTACTATGAAGGATTCTGAGTACTTTAAGAGTAAGTTCAAACACCTACTCAAAATGGTCAGAGAGAGGCACAGAGTATGCCTGGCTAAGTACTATCTGAACAGTCCGTTCAGAGAGCTTTTGGAAAGGAAGGCGCTCAGGAAAGAGTGGCTTCGAGCTGAATCGTCTGTGTTGTAGAGCGTATTGCTCTCGAATCAGAAGACTAGGAGTGATGAAGTTGAGCAATCATCACGCCATATGAAAAAGCTTAAAACTTCGCGTTAAAAAATAATACGCGATTTTAAGAGGGGGATGATTACCAATTGTGGCCTCCTGACACTTAAGAATTATCAC
