# Supplementary figures and images for: Characterization and complete genome sequences of two novel variants of the family Closteroviridae from Chinese kiwifruit
Source: PLoS One. 2020 Nov 23;15(11):e0242362. doi: 10.1371/journal.pone.0242362 (PMC7682855; doi:10.1371/journal.pone.0242362)

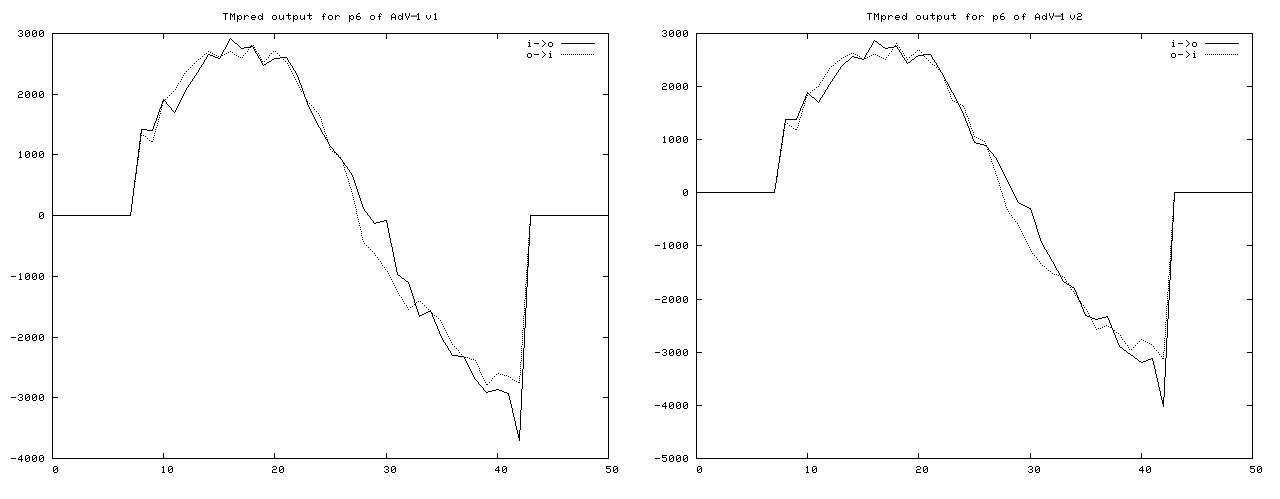

Supplement: S3 Fig — (JPG) [file pone.0242362.s004.jpg]
