# Supplementary material for: Characterization and complete genome sequences of two novel variants of the family Closteroviridae from Chinese kiwifruit
Source: PLoS One. 2020 Nov 23;15(11):e0242362. doi: 10.1371/journal.pone.0242362 (PMC7682855; doi:10.1371/journal.pone.0242362)
Supplement: S1 Table — (DOC) [file pone.0242362.s006.doc]

**S1 Table. Oligonucleotide primers used for RACE-PCR of *Actinidia* *deliciosa* *virus* 1 variants**

| Gene name | Primer sequence (5′-3′) | Tm /°C | Size /bp | Purpose |
| --- | --- | --- | --- | --- |
| AdV-1 variant 1 | V1Race-5R1:CACATAGGCGACTGGAAAGGTG | 57 | 826 | 1^st^ round of 5′-RACE |
|  | V1Race-5R2: AGCACTCTCCCTTGATGTTGAG | 57 | 377 | 2^nd^ round of 5′-RACE |
|  | V1Race-3F1: ACTTCATTATCCAGGCAGCCCACG | 59 | 505 | 1^st^ round of 3′-RACE |
|  | V1Race-3F2: GGAAACCCATCCAAACCCTGAGAG | 60 | 477 | 2^nd^ round of 3′-RACE |
| AdV-1 variant 2 | V2Race-5R1: GTAGGAAGGCTTCAAAGTGGTAC | 55 | 768 | 1^st^ round of 5′-RACE |
|  | V2Race-5R2: ACGATCCAACCTCCCACGCTC | 59 | 685 | 2^nd^ round of 5′-RACE |
|  | V2Race-3F1: CACTAATGTGGTGGACAACGCTAAG | 58 | 703 | 1^st^ round of 3′-RACE |
|  | V2Race-3F2: CTATGTACGAGGATATGATGCGAC | 55 | 434 | 2^nd^ round of 3′-RACE |
| Universal primer | 10UPM:CTAATACGACTCACTATAGGGCAAGCAGTGGTATCAACGCAGAGT | - | - | 1^st^ round of 5′/3′-RACE |
|  | NUP: AAGCAGTGGTATCAACGCAGAGT | - | - | 2^nd^ round of 5′/3′-RACE |
