# Supplementary material for: Characterization and complete genome sequences of two novel variants of the family Closteroviridae from Chinese kiwifruit
Source: PLoS One. 2020 Nov 23;15(11):e0242362. doi: 10.1371/journal.pone.0242362 (PMC7682855; doi:10.1371/journal.pone.0242362)
Supplement: S2 Table — (DOC) [file pone.0242362.s007.doc]

**S2 Table. Sequence identities of *Actinidia deliciosa virus* 1 variant 1 and 2 with other members of the family *Closteroviridae* analyzed by ClustalX**

| **Genus** | **GenBank no.** | **Virus and Acronym** | **Nucleotide sequence identity (%)^a^** | **Amino acid sequence identity (%)^b^** | | | |
| --- | --- | --- | --- | --- | --- | --- | --- |
|  |  |  |  | **ORF1a** | **RdRp** | **Hsp70h** | **CP** |
| Unassigned | - | Actinidia deliciosa virus1 variant2（AdV-1 v2） | 62.80 | 60.11 | 80.47 | 88.87 | 88.07 |
| Unassigned | KX857665 | Actinidia virus 1(AcV-1) | 81.38 | 86.50 | 93.53 | 93.15 | 93.42 |
| Unassigned | NC025967 | Persimmon virus B variant 1 (PeVB v1) | 42.26 | 18.35 | 37.94 | 33.28 | 20.88 |
| Unassigned | AB923925 | Persimmon virus B variant 2 (PeVB v2) | 42.09 | 18.65 | 36.65 | 34.29 | 21.61 |
| Closterovirus | BYU51931 | Beet yellow stunt virus (BYSV) | 27.12 | 5.95 | 33.92 | 26.66 | 18.03 |
| Closterovirus | NC006944 | Mint virus 1 (MV-1) | 38.06 | 16.57 | 34.97 | 26.14 | 17.28 |
| Closterovirus | KU883267 | Citrus tristeza virus (CTV) | 40.85 | 18.68 | 33.40 | 28.29 | 19.34 |
| Closterovirus | NC027712 | Tobacco virus 1 (ToV-1) | 38.38 | 15.99 | 33.40 | 27.48 | 16.80 |
| Closterovirus | NC_008585 | Raspberry mottle virus (RMV) | 40.76 | 14.93 | 34.84 | 28.29 | 19.34 |
| Crinivirus | NC010560, EU191905 | Bean yellow disorder virus (BYDV) | - | 11.59 | 21.88 | 23.78 | 17.37 |
| Crinivirus | NC005209, NC005210 | Beet pseudo-yellows virus（BPYV） | - | 12.15 | 23.48 | 24.66 | 14.45 |
| Ampelovirus | AF414119 | Pineapple mealybug wilt-associated virus 1 (PMWaV-1) | 34.01 | 12.24 | 24.83 | 21.98 | 17.36 |
| Ampelovirus | NC016509 | Grapevine leafroll-associated virus 1 (GLRaV-1) | 41.15 | 12.59 | 22.77 | 21.94 | 14.50 |
| Velarivirus | HM588723 | Cordyline virus 1 (CoV-1) | 42.05 | 13.29 | 22.94 | 23.27 | 13.61 |
| Velarivirus | HE588185 | Grapevine leafroll-associated virus 7(GLRaV-7) | 41.91 | 13.55 | 21.45 | 25.13 | 13.77 |

a. Nucleotide sequence alignment was performed using ClustalX software using complete alignment with default parameters.

b. Amino acid sequence alignment was performed using ClustalX software using complete alignment with default parameters.
