# Supplementary material for: Characterization and complete genome sequences of two novel variants of the family Closteroviridae from Chinese kiwifruit
Source: PLoS One. 2020 Nov 23;15(11):e0242362. doi: 10.1371/journal.pone.0242362 (PMC7682855; doi:10.1371/journal.pone.0242362)
Supplement: S3 Table — (DOC) [file pone.0242362.s008.doc]

**S3 Table. Molecular weights (kDa) of proteins encoded by the ORFs of the AdV-1 variants and AcV-1.**

| Virus name | ORF1  （ORF1a） | ORF2  (RdRp) | ORF3 | ORF4 | ORF5 | ORF6  (Hsp70h) | ORF7 | ORF8 | ORF9  (CP) | ORF10 | ORF11 | ORF12 |
| --- | --- | --- | --- | --- | --- | --- | --- | --- | --- | --- | --- | --- |
| AdV-1 v1 | 356.71 | 58.29 | 13.68 | 25.56 | 5.58 | 64.12 | 58.80 | 27.53 | 27.34 | - | - | - |
| AdV-1 v2 | 191.84 (ORF1a1),  160.59 (ORF1a2) | 58.90 | 14.34 | 25.83 | 5.67 | 64.60 | 27.25 (ORF7a),  29.29 (ORF7b) | 18.39 (ORF8a),  10.00 (ORF8b) | 27.12 | 18.45 | 7.58 | 22.35 |
| AcV-1 | 356.58 | 58.63 | 13.57 | 25.42 | 5.62 | 64.10 | 59.06 | 29.86 | 27.39 | 18.64 | 7.57 | 15.49 |
