# Supplementary material for: Characterization and complete genome sequences of two novel variants of the family Closteroviridae from Chinese kiwifruit
Source: PLoS One. 2020 Nov 23;15(11):e0242362. doi: 10.1371/journal.pone.0242362 (PMC7682855; doi:10.1371/journal.pone.0242362)
Supplement: S5 Table — (DOC) [file pone.0242362.s010.doc]

**S5 Table.** Lengths (nt/aa) of nucleotide sequences and protein sequences of the ORFs of AdV-1 variants and AcV-1

| Virus name | ORF1  (ORF1a) | ORF2  (RdRp) | ORF3 | ORF4 | ORF5 | ORF6  (Hsp70h) | ORF7 | ORF8 | ORF9  (CP) | ORF10 | ORF11 | ORF12 |
| --- | --- | --- | --- | --- | --- | --- | --- | --- | --- | --- | --- | --- |
| AdV-1 v1 | 9558/3185 | 1524/507 | 339/112 | 675/224 | 156/51 | 1755/584 | 1524/507 | 738/245 | 732/243 | - | - | - |
| AdV-1 v2 | 5208/1735 (ORF1a1),  4332/1443 (ORF1a2) | 1524/507 | 366/121 | 675/224 | 156/51 | 1755/584 | 708/235 (ORF7a),  756/251 (ORF7b) | 489/162 (ORF8a),  270/89 (ORF8b) | 732/243 | 474/157 | 213/70 | 576/191 |
| AcV-1 | 9558/3185 | 1533/510 | 339/112 | 675/224 | 156/51 | 1755/584 | 1524/507 | 798/265 | 732/243 | 474/157 | 213/70 | 405/134 |
